# Metabolic Alterations in Colombian Women with Rheumatoid Arthritis and Systemic Lupus Erythematosus Reveal Potential Lipid Biomarkers Associated with Inflammation and Cardiovascular Risk

**DOI:** 10.3390/ijms26104527

**Published:** 2025-05-09

**Authors:** Nancy Paola Duarte-Delgado, Juan Manuel Bello-Gualtero, Daniel G. Fernández-Ávila, Consuelo Romero-Sánchez, Stefano Cacciatore, Mónica P. Cala, Luz-Stella Rodríguez Camacho

**Affiliations:** 1 Instituto de Genética Humana, Facultad de Medicina, Pontificia Universidad Javeriana, Carrera 7 # 40-62, Bogotá 110231, Colombia; duarte.np@javeriana.edu.co; 2 Bioinformatics Unit, International Centre for Genetic Engineering and Biotechnology (ICGEB), Rooms 219 Werhner and Beit South UCT Campus, Anzio Road, Observatory, Cape Town 7925, South Africa; stefano.cacciatore@icgeb.org; 3 Grupo de Inmunología Clínica Aplicada, Servicio de Reumatología, Hospital Militar Central, Facultad de Medicina, Universidad Militar Nueva Granada, Tv. 3C No. 49-02, Bogotá 110111, Colombia; juanmabello36@gmail.com (J.M.B.-G.); romeromaria@unbosque.edu.co (C.R.-S.); 4 Unidad de Reumatología, Hospital Universitario San Ignacio, Carrera 7 # 40-62, Bogotá 110231, Colombia; daniel.fernandez@javeriana.edu.co; 5 Departamento de Medicina Interna, Pontificia Universidad Javeriana, Carrera 7 # 40-62, Bogotá 110231, Colombia; 6 Grupo InmuBo, Universidad El Bosque, Ak. 9 #131a-2, Bogotá 111321, Colombia; 7 Metabolomics Core Facility-MetCore, Universidad de los Andes, Ak. 9 #131a-2, Bogotá 111711, Colombia; mp.cala10@uniandes.edu.co

**Keywords:** cytokines, HDL, metabolites, rheumatoid arthritis, systemic lupus erythematosus

## Abstract

Rheumatoid arthritis (RA) and systemic lupus erythematosus (SLE) are autoimmune diseases associated with chronic inflammation and cardiovascular risk. This study aimed to identify metabolic alterations in Colombian women with RA and SLE to discover potential biomarkers. Plasma samples were analyzed using LC-QTOF-MS and GC-QTOF-MS. Correlation network analysis assessed relationships between metabolites, cytokines, and HDL levels. A generalized linear model (GLM) combined metabolite scores, and ROC analysis evaluated their predictive performance. Significant metabolic changes were observed, including decreased phospholipids and sphingolipids, and increased glycerolipids in RA and SLE compared to healthy controls. The metabolite–cytokine network revealed correlations between FA 18:0 and DG 37:7 with cytokines, linking lipid metabolism to inflammation. PS O-40:3 and FA 18:0 in RA and PC O-28:0 and DG 37:7 in SLE distinguished patients from healthy controls. The combination of PS O-40:3 and FA 18:0 in RA (AUC = 0.997) and PC O-28:0 and DG 37:7 in SLE (AUC = 0.949) demonstrated high predictive performance. PE O-42:5 was positively correlated with HDL, suggesting a potential protective role against cardiovascular disease. These findings highlight lipid metabolism’s role in RA and SLE and support specific metabolites as biomarkers for disease differentiation, inflammation, and cardiovascular risk. These insights could lead to improved diagnostics and targeted treatments for these autoimmune diseases.

## 1. Introduction

Rheumatoid arthritis (RA) and systemic lupus erythematosus (SLE) are chronic autoimmune diseases characterized by the loss of tolerance to self-cellular components [1]. RA primarily affects joints with autoantibodies targeting citrullinated peptides, while SLE involves multiple organs and autoantibodies against nuclear and cytoplasmic antigens [2,3]. Both RA and SLE share immunological features such as altered immune cell composition and dysregulated gene expression profiles, particularly involving T cells, B cells, and monocytes. Recent transcriptomic analyses have identified shared functional subtypes and overlapping molecular mechanisms in both diseases [4].

RA and SLE are caused by the interaction of genetic and environmental factors that, over time, induce disease evolution from genetic risk to the autoimmune disease phenotype meeting diagnostic or classification criteria [5]. The resemblance in their underlying pathophysiology, along with the shared genetic and environmental triggers, suggests a common origin for these diseases, often referred to as the autoimmune tautology [6]. The main genetic influence has been identified at the major histocompatibility complex (MHC) involved with antigen presentation, but loci involved with lymphocyte activation and intracellular signaling like PTPN22, CTLA4, and STAT4 have also been associated [7]. The environmental factors are less well studied but include diet, xenobiotics, pollution, infections, and personal lifestyles [5].

Given the complexity of these interactions, “omics” disciplines, particularly metabolomics, have been introduced to provide a comprehensive approach to understanding the pathophysiology of multifactorial diseases like RA and SLE [2,8,9]. Metabolomics measures the complete set of metabolites in a biological sample, providing insight into disease mechanisms by studying metabolic alterations [10,11]. The field of immunometabolism has emerged, highlighting the role of metabolism in immune cell differentiation and function, contributing to autoimmune disease development [12,13,14,15].

RA prevalence in Colombia is 240 per 100.000 inhabitants [16] and for SLE, it is 91.9 per 100.000 inhabitants [17]. Therefore, RA and SLE have a prevalence that is higher than in other Latin American countries and have become a public health problem due to the progressive disability experienced by patients and the high socioeconomic cost they represent [18]. The unpredictability of these diseases, with periods of remission and relapse, makes monitoring and treatment difficult. Although biomarkers such as C-reactive protein (CRP) in RA and serum complement levels in SLE are widely used in clinical practice, they present important limitations. In RA, CRP levels may not consistently reflect disease activity, particularly in patients with low or moderate disease activity, or in those undergoing treatment that suppresses CRP expression [19]. In SLE, complement proteins such as C3 and C4 may be influenced by genetic and environmental factors, and their levels do not always correlate with disease activity or organ involvement [20]. Hence, there is a need for the development of reliable biomarkers to diagnose these patients, aiming to enhance their well-being and sustain their remission.

The exploration of biomarkers has traditionally focused on immune molecules, and “omics” techniques, including metabolomics, have been utilized to identify novel diagnostic and therapeutic targets [21,22]. Previous reviews have shown an overlap in metabolic pathways between RA and SLE, consistent with their shared pathophysiology. Notably, SLE is marked by altered tryptophan metabolism, leading to increased kynurenine, while RA shows up-regulation of the pentose phosphate pathway, reflected in elevated ribose levels [23].

Integrating diverse data types, including metabolomics, cytokine profiles, and clinical data, is crucial for understanding disease pathophysiology and identifying robust biomarkers [24]. Our study identified lipid biomarkers linked to inflammation, oxidative stress, and cardiovascular risk in RA and SLE using untargeted metabolomics, uncovering significant correlations with cytokine and HDL levels. These findings could lead to improved diagnosis and targeted treatment strategies, potentially improving patient outcomes. This discovery sheds light on the pathophysiologic mechanisms of these diseases and has important clinical implications, potentially leading to better patient outcomes.

## 2. Results

### 2.1. Characteristics of the Participants

We analyzed the metabolic profiles of plasma samples from 72 Colombian women, including 23 with RA, 22 with SLE, and 27 age- and BMI-matched HCs. Clinical and demographic characteristics are detailed in Table 1. The median ages for RA, SLE, and HC groups were 43, 35, and 40 years, respectively. SLE patients had a higher median disease duration (5.82 years) than RA patients (4.53 years), though the difference was not statistically significant. Disease activity scores were a median of 3.6 for DAS28-ESR (RA) and 6 for SLEDAI (SLE). Statistically significant differences in lymphocyte and neutrophil counts were found for RA vs. HC and SLE vs. HC, respectively, with lymphocyte counts lower and neutrophil counts higher in both diseases.

Dyslipidemia was evident, with RA and SLE patients showing altered lipid profiles, including elevated LDL, triglycerides, and total cholesterol, alongside decreased HDL levels [25,26]. A significant reduction in HDL was observed only in SLE (Table 1). Low HDL was found in 30.4% of RA and 31.8% of SLE patients (Table S1). Despite higher median LDL levels in HCs (Table 1), high LDL was present in 52.2% of RA and 63.6% of SLE patients. Hypercholesterolemia was noted in 30.4% of RA and 22.7% of SLE patients, while triglycerides were elevated in 39.1% of RA and 31.8% of SLE patients (Table S1). Serological characterization showed that most rheumatoid arthritis (RA) patients were positive for rheumatoid factor (RF), consistent with seropositive RA. Among systemic lupus erythematosus (SLE) patients, 28.6% tested positive for anti-double-stranded DNA (anti-dsDNA) antibodies (Table 1). Additionally, complement abnormalities were common in SLE: 77.3% of patients had low C3 levels, while 22.7% showed low C4 levels (Table S1). Treatment regimens varied, with methotrexate (65.2%) and oral glucocorticoids (69.6%) being predominant in RA, while antimalarials (87%) and glucocorticoids (45.5%) were common in SLE (Table S1). The majority of rheumatoid arthritis (RA) patients (93.8%) and systemic lupus erythematosus (SLE) patients (70.0%) received an oral glucocorticoid (GC) dose of ≤15 mg, indicating that lower doses were the most commonly used in both groups (Table S1). Given that higher GC doses are associated with more pronounced metabolic disturbances, the metabolic effects in these patients are expected to be less pronounced with this dose.

### 2.2. Metabolite Mapping of RA and SLE Patients

**Figure 1:**
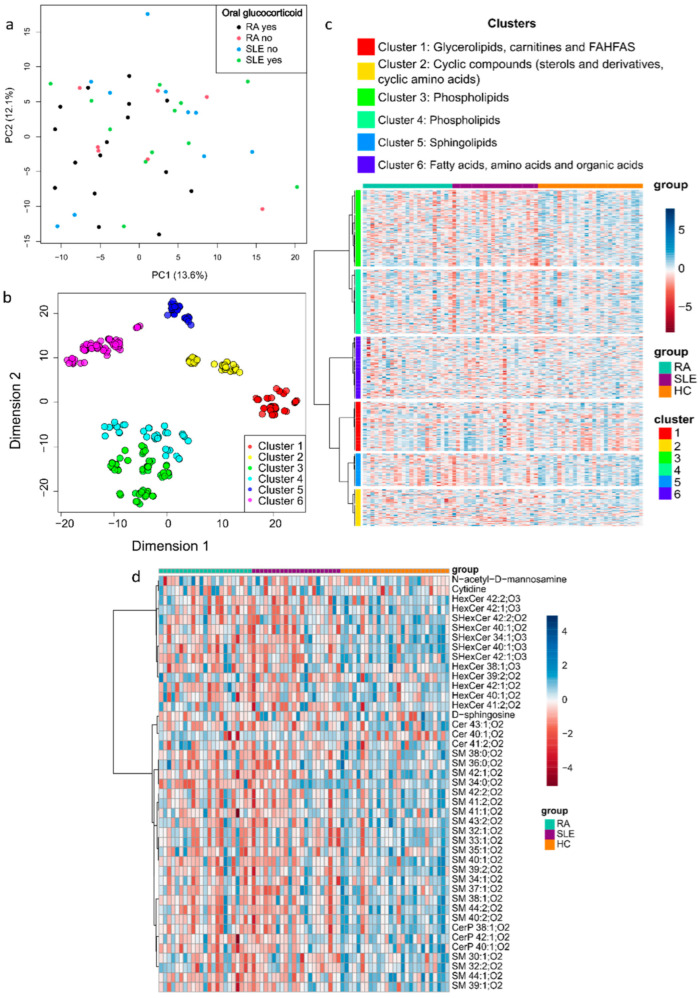
Metabolite mapping of RA patients, SLE patients, and HCs. (**a**) PCA was performed to assess the impact of medication on the metabolic profiles of RA and SLE patients. (**b**) The metabolites were classified according to their chemical similarity in six clusters. Each colored circle represents a metabolite. (**c**) Heatmap showing the variation of the metabolites belonging to each cluster across the study groups. (**d**) Heatmap showing in detail the variation of the metabolites belonging to cluster 5 (sphingolipids).

After processing the metabolomics data, we obtained the relative levels of 431 metabolites. The information regarding the chemical formula, mass, retention time (RT), %CV QC, and confidence level of identification is in Table S2. Furthermore, the data regarding the *p*-values, FDR, fold change, and VIP values for all the metabolites can be found in Table S3. The PCA in Figure 1a showed that the metabolic profiles of RA and SLE patients were not significantly influenced by medication use, as patients taking or not taking oral glucocorticoids did not form distinct clusters. Metabolites were categorized into six clusters based on chemical properties.

Applying the MetChem package enabled the classification of metabolites into six clusters based on chemical similarity. Figure 1b presents these clusters in a two-dimensional space, highlighting cluster 1 (glycerolipids, carnitines, and FAHFAs), cluster 2 (cyclic compounds, sterols, and cyclic amino acids), clusters 3 and 4 (phospholipids), cluster 5 (sphingolipids), and cluster 6 (fatty acids, amino acids, and organic acids). The heatmap in Figure 1c provides an overview of compound variation, revealing a decrease in phospholipids (clusters 3 and 4) and sphingolipids (cluster 5) in RA and SLE compared to HCs. A detailed view of the variation in the cluster of sphingolipids is shown in Figure 1d, where most compounds were reduced in RA and SLE. The heatmaps showing the variation in the other clusters are available in Supplementary Figure S3.

**Figure 2:**
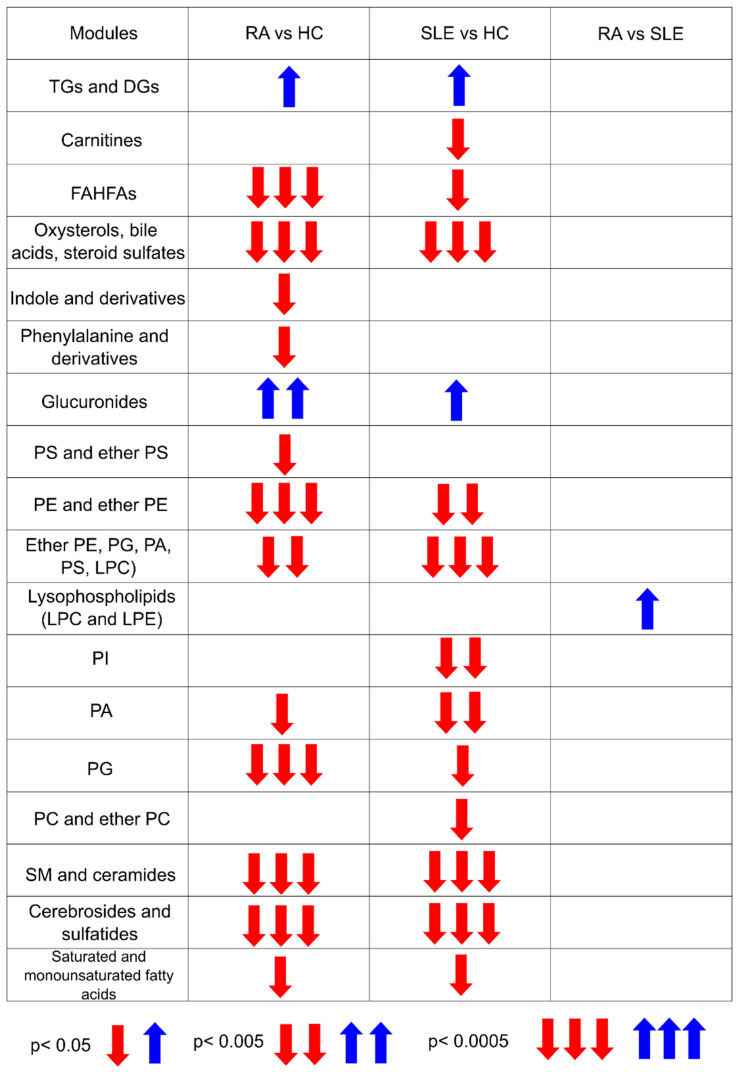
The significance levels obtained from Dunn’s test for multiple comparisons with Bonferroni correction of the metabolite alterations are presented.

The compounds within each cluster were further grouped into modules based on hierarchical clustering. The WMCSA function was used to summarize relative metabolite levels, which are visualized in the heatmaps in Figure S4, highlighting inter-group differences across the six previously identified clusters. Notably, most metabolic alterations were observed in lipid classes. The statistical significance of these module comparisons is summarized in Figure 2.

Phospholipids, key components of cell membranes, consist of a glycerol backbone ester bonded to two fatty acids and a phosphate group, which is linked to a polar group. These phospholipids are categorized into various classes, such as phosphatidylcholine (PC), phosphatidylethanolamine (PE), phosphatidylserine (PS), phosphatidylinositol (PI), phosphatidic acid (PA), and phosphatidylglycerol (PG), with PC and PE being the most abundant in mammalian cells [27]. Across the RA and SLE patient groups, significant variations in phospholipid levels were observed compared to HCs (Figure S4c). PE, including ether PEs, as well as PAs and PGs, were decreased in both RA and SLE, while PSs, including ether PSs, were specifically decreased in RA versus HC, and PIs were significantly reduced in SLE versus HC. Ether phospholipids, which have an alkyl or vinyl bond linking a fatty alcohol at the sn-1 position of the glycerol backbone [28], were also significantly decreased in RA and SLE compared to HC. Lysophospholipids, characterized by having only one fatty acid moiety, were significantly increased in RA compared to SLE, representing the only significant difference between the two diseases. Additionally, PCs, including ether PCs, were significantly decreased in SLE compared to HCs (Figure S4d).

Sphingolipids, which are composed of a sphingosine backbone, showed further alterations. Ceramides, which act as pro-apoptotic signals, and sphingomyelins (SMs), which form lipid raft domains in cell membranes, were significantly decreased in RA and SLE compared to HCs (Figure S4e) [27,29,30]. Fatty acids, which serve as energy sources and precursors to bioactive and membrane lipids [27], were also significantly reduced in RA and SLE compared to HCs (Figure S4f). Glycerolipids, represented by triglycerides (TGs) and diglycerides (DGs), were significantly increased in RA and SLE compared to HCs. Plasma TG concentration is a biomarker for TG-rich lipoproteins in circulation [30], while DGs play a key role in membranes and as secondary messengers [31]. Carnitines were significantly decreased only in SLE compared to HCs (Figure S4a). Lastly, non-lipid compounds, such as phenylalanine and indole derivatives, were significantly decreased in RA compared to HC (Figure S4b).

### 2.3. Metabolite and Cytokine Correlation Network in RA and SLE

In our RA and SLE patients, we observed significant increases in several cytokines, including GM-CSF, CX3CL1, IFN-α2, IL-12p70, IL-17A, TNF-α, IL-1β, and IFN-γ in both diseases, while MCP-1 and IL-10 were exclusive to SLE, and IL-2 was specific to RA [32]. To explore relationships between metabolites and cytokines, we performed correlation network analyses.

**Figure 3:**
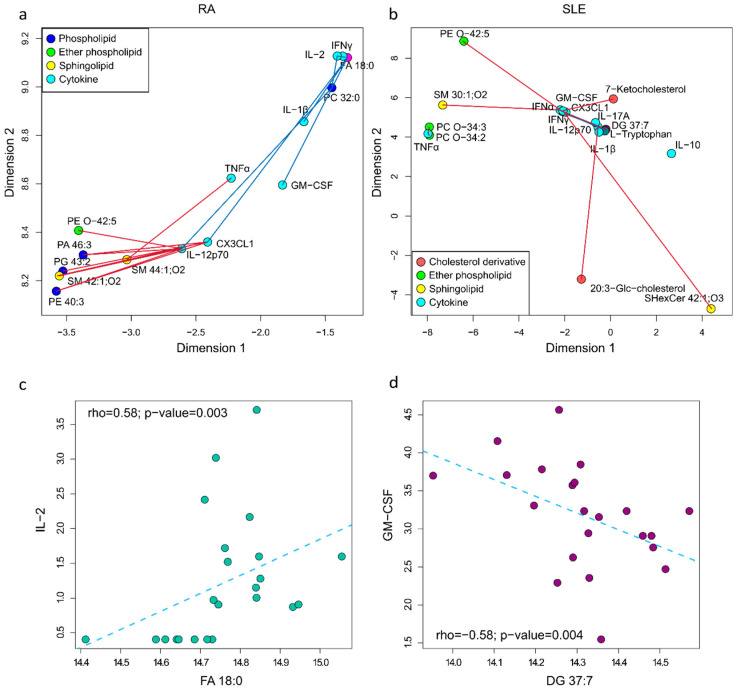
Metabolite and cytokine correlation network in (**a**) RA patients and (**b**) SLE patients. Positive and negative correlations are represented by blue and red lines, respectively. (**c**) Correlation between IL-2 and FA 18:0 in RA patients. (**d**) Correlation between GM-CSF and DG 37:7 in SLE patients.

For RA (Figure 3a), we found negative correlations between sphingomyelins (SM 42:1;O2 and SM 44:1;O2), phospholipids (PA 46:3, PG 43:2, PE 40:3), and the ether phospholipid (PE-O 42:5) with IL-12p70, CX3CL1, and TNFα. A positive correlation between FA 18:0 and four cytokines (GM-CSF, CX3CL1, IL-2, IL-12p70) was notable. The positive correlation between FA 18:0 and IL-2 is shown in Figure 3c. The rho and *p*-value of these correlations can be found in Table S4a.

In SLE (Figure 3b), fewer correlations were identified, and were mostly negative, including correlations between ether phospholipids (PE O-42:5, PC O-34:3, and PC O-34:2), sphingolipids (SM 30:1;O2, SHexCer 42:1;O3), and cholesterol derivatives with pro-inflammatory cytokines like IFNα, IFNγ, TNFα, IL-12p70, CX3CL1, and GM-CSF. The only positive correlation was between L-tryptophan and CX3CL1, while DG 37:7 showed negative correlations with CX3CL1 and GM-CSF. The negative correlation between DG 37:7 and GM-CSF is shown in Figure 3d. The rho and *p*-value of all these correlations can be found in Table S4b.

Interestingly, the lipid PE O-42:5 was found to be significantly correlated with cytokines in both RA and SLE. In RA (Figure S5a), the negative correlation with IL-12p70 is shown, while in SLE (Figure S5b), its negative correlation with GM-CSF indicates ether phospholipids’ involvement in inflammatory processes in both diseases.

### 2.4. Predictive Performance of Combined Metabolites in RA and SLE

**Figure 4:**
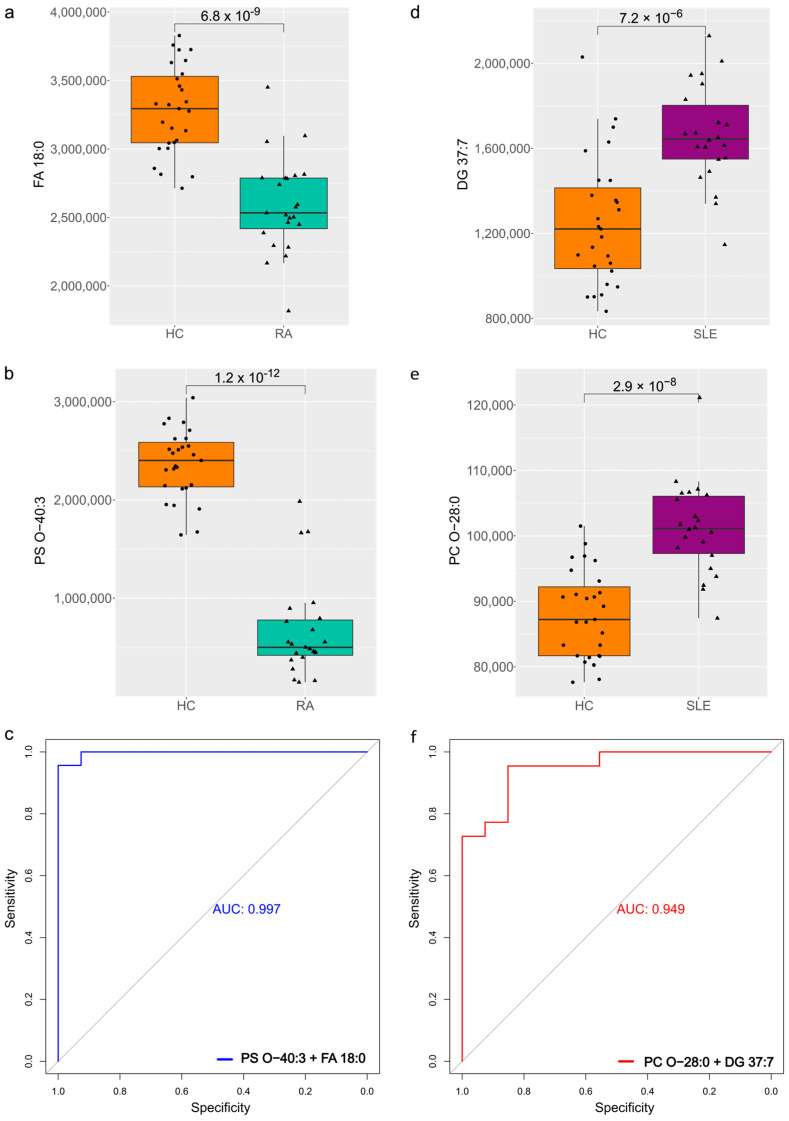
(**a**) Box plot of FA 18:0 levels across HC and RA. (**b**) Box plot of PS O-40:3 levels across HC and RA. (**c**) Receiver operating characteristic (ROC) curve for combined biomarker PS O-40:3 + FA 18:0. (**d**) Box plot of DG 37:7 levels across HC and SLE. (**e**) Box plot of PC O-28:0 levels across HC and SLE. (**f**) Receiver operating characteristic (ROC) curve for combined biomarker PC O-28:0 and DG 37:7.

FA 18:0 and DG 37:7 emerged as important players in the metabolite–cytokine networks for RA and SLE, respectively, due to their significant correlations with cytokines. The boxplots in Figure 4a show that FA 18:0 levels were significantly lower in RA compared to HCs, while Figure 4b illustrates that DG 37:7 was significantly higher in SLE compared to HCs. Given these differences, we aimed to assess their predictive value in combination with PS O-40:3 for RA and PC O-28:0 for SLE, as these metabolites exhibited the strongest statistical significance in their respective disease comparisons. As seen in Figure 4c,d, PS O-40:3 was significantly reduced in RA vs. HC, while PC O-28:0 was significantly lower in SLE vs. HC.

A binomial generalized linear model (GLM) was applied to combine the values of the two selected metabolites for each disease. This model estimates the probability of a sample belonging to the RA or SLE group by computing a linear combination of the metabolite concentrations weighted by their respective regression coefficients. The predicted probabilities were then used to generate ROC curves. The model demonstrated high classification performance, achieving an AUC of 0.997 for RA (PS O-40:3 + FA 18:0, Figure 4e) and 0.949 for SLE (PC O-28:0 + DG 37:7, Figure 4f), indicating a strong discriminatory power between disease and healthy states.

### 2.5. Metabolite and HDL Correlation Network

**Figure 5:**
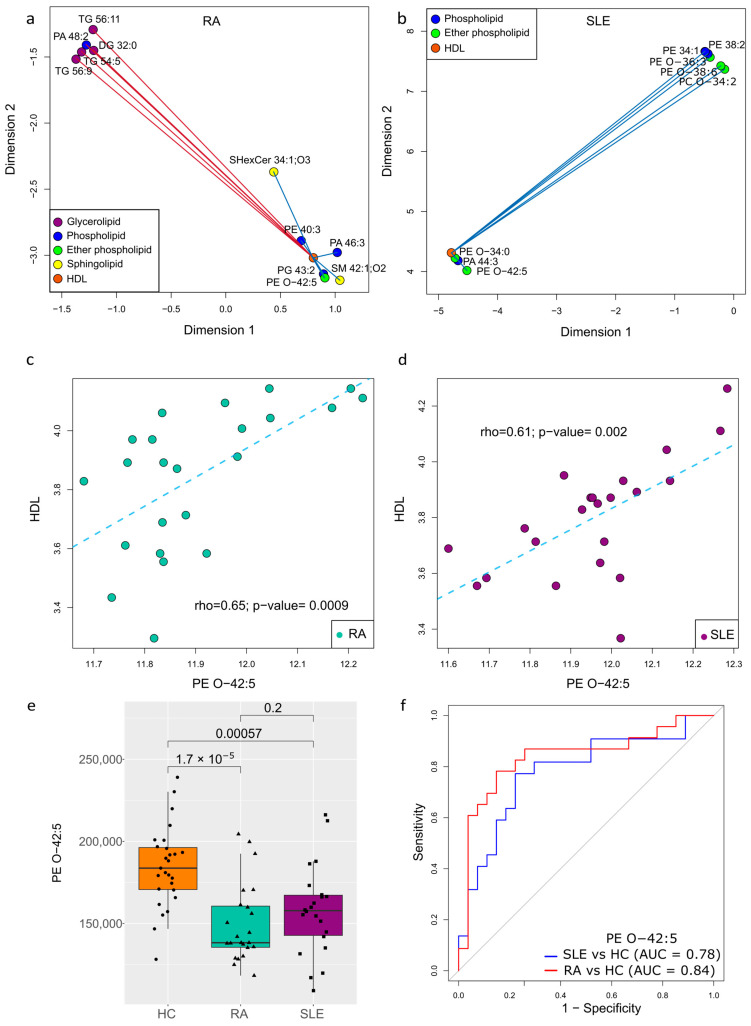
PE O-42:5 levels distinguish RA from HC and SLE from HC, and correlate with HDL (**a**) Box plot of PE O-42:5 levels across HC, RA, and SLE groups. (**b**) Metabolite and HDL correlation network in RA. (**c**) Metabolite and HDL correlation network in SLE. Positive and negative correlations are represented by blue and red lines, respectively. Correlation between PE O-42:5 in (**c**) RA and (**d**) SLE patients. (**e**) Box plot of PE O-42:5 levels across HC, RA, and SLE. (**f**) ROC curves for PE O-42:5 levels in RA vs. HC and SLE vs. HC.

Given the decrease in HDL levels observed in RA and SLE patients compared to HCs (Table 1), we aimed to establish the relationships between this lipid profile parameter and the significantly altered metabolites in RA and SLE, most of which were lipids. In the metabolite–HDL network for RA, we observed a set of negative correlations, primarily with glycerolipids (TG 56:9, TG 54:5, DG 32:0, and TG 56:11), and positive correlations with phospholipids (PE 40:3, PA 46:3, and PG 43:2), sphingolipids (SM 42:1;O2 and SHexCer 34:1;O3), and an ether phospholipid (PE O-42:5) (Figure 5a). The rho and *p*-values for these correlations are presented in Table S5a.

For SLE, the correlation network revealed only positive correlations with phospholipids (PE 38:2 and PA 44:3) and ether phospholipids (PE O-36:3, PE O-38:6, PC O-34:2, PE O-34:0, and PE O-42:5) (Figure 5b), with detailed rho and *p*-values available in Table S5b. Since PE O-42:5 showed a significant positive correlation with HDL in both RA and SLE, we examined this relationship in greater detail. Figure 5c illustrates this significant correlation in RA, while Figure 5d demonstrates the same in SLE. Given that PE O-42:5 was significantly reduced in both RA and SLE compared to HCs (Figure 5e), and considering its positive association with HDL, this ether phospholipid may play a protective role against cardiovascular disease (CVD) risk.

To further assess its potential clinical relevance, we performed an ROC analysis to evaluate its diagnostic performance. The results demonstrated an AUC of 0.84 for RA vs. HC, which outperformed the 0.78 observed for SLE vs. HC (Figure 5f), reinforcing the potential role of PE O-42:5 as a biomarker linked to lipid metabolism and cardiovascular risk in these diseases.

## 3. Discussion

This study highlights metabolic alterations in RA and SLE, particularly in lipid metabolism. Both diseases showed decreased phospholipids and sphingolipids, with increased glycerolipids. Some patients exhibited dyslipidemia, characterized by high LDL and total cholesterol with low HDL, potentially worsened by glucocorticoids, though methotrexate and hydroxychloroquine may counteract these effects [26]. Elevated triglycerides (TGs) align with dyslipidemia in SLE [33], and a high TG/low HDL profile is linked to systemic inflammation and poor TNFα blockade response [34]. Increased diglycerides (DGs), key in cell signaling [31], were also noted.

Phospholipid decreases, particularly phosphatidylinositols (PIs), likely reflect increased membrane turnover and signaling demands during inflammation [35]. PI reduction in SLE compared to HC could be related to its consumption in inflammatory signaling pathways [36]. Increased lysophospholipids, known to act as signaling molecules and byproducts of phospholipase A2 activity, were notably higher in RA [37]. A Chinese SLE cohort also reported increased lysophospholipids [38]. Ether phospholipids, reduced in RA and SLE, are important antioxidants, and their depletion is linked to oxidative stress, as previously reported in SLE [38]. Sphingolipids, essential in cell membrane structure and immune signaling [29,39], were decreased in RA and SLE, likely due to their increased demand for lipid raft formation and immune cell activation.

The metabolite–cytokine network revealed correlations between sphingolipids, ether phospholipids, and cytokines in both RA and SLE, highlighting the role of oxidative stress and lipid raft disruption in inflammation [40]. Oxidative stress activates NFκB, promoting pro-inflammatory cytokine production (IFNγ, TNFα, IL-1) and linking lipid metabolism to immune dysregulation and CVD risk [26,41]. In RA, FA 18:0 positively correlated with GM-CSF, CX3CL1, IL-2, and IL-12p70, consistent with its role in inflammatory responses in fibroblast-like synoviocytes [40,42], though contrasting reports suggest a negative correlation with disease activity [43]. In SLE, DG 37:7 was negatively correlated with CX3CL1 and GM-CSF, likely reflecting its role in macrophage-driven inflammatory regulation [44].

Ether phospholipids like PS O-40:3 and PC O-28:0, involved in antioxidative defense and membrane stability, may contribute to increased oxidative stress when reduced, potentially worsening RA and SLE progression through lipid peroxidation and impaired inflammation resolution [41]. The combined metabolite score of PS O-40:3 and FA 18:0 (Figure 4e) demonstrates their potential as diagnostic markers for distinguishing RA from healthy controls (HC). Similarly, the combination of PC O-28:0 and DG 37:7 (Figure 4f) highlights their strong diagnostic performance in differentiating SLE from HC, emphasizing the value of specific lipid biomarkers in disease classification.

The metabolite–HDL network revealed negative correlations between HDL and TGs in RA, suggesting a protective role against CVD risk. Lipoprotein alterations, oxidative stress, and lipid peroxidation contribute to the heightened CVD susceptibility in RA and SLE [45]. Reduced ether phospholipids, particularly plasmalogens in HDL, have been linked to endothelial apoptosis, exacerbating cardiovascular risks [46]. These findings emphasize the need to investigate lipid abnormalities, including HDL composition, in disease-related CVD risk. Additionally, PE O-42:5 was identified as a protective marker against CVD in RA and SLE, reinforcing the relevance of lipidomic studies.

Additionally, the correlation between PE O-42:5 and cytokines in RA and SLE underscores the relationship between oxidative stress and inflammation in both diseases. This connection is particularly relevant to the pathophysiology of atherosclerosis, a major risk factor for CVD. Atherosclerosis is driven by chronic inflammation and oxidative stress, both of which promote endothelial dysfunction, lipid accumulation, and plaque formation. Therefore, these findings illustrate how pathophysiological mechanisms such as oxidative stress and inflammation are interconnected, contributing to the increased CVD risk observed in RA and SLE patients.

While the sample size may affect statistical power, further validation in larger cohorts is needed. Recruiting untreated RA and SLE patients is challenging due to the necessity of ongoing treatment, but PCA suggests that medication did not significantly influence metabolic profiles (Figure 1a and Figure S6). However, future longitudinal studies are needed to follow patients from diagnosis onward. To ensure robust comparisons with healthy controls, we aimed to analyze a homogeneous population of SLE and RA patients. The exclusion of patients with antiphospholipid syndrome, neuropsychiatric SLE, stage IV/V lupus nephritis, or recent rituximab treatment helped minimize confounding factors associated with severe disease manifestations and treatment effects. This approach allowed us to detect strong metabolic differences without the variability introduced by distinct SLE subgroups, which could have made it more challenging to identify differential metabolites. This was a methodological decision, but we acknowledge that this selection criterion restricts the generalizability of our findings to all SLE subtypes. Despite these limitations, the study highlights promising lipid biomarkers linked to inflammation, oxidative stress, and CVD risk in RA and SLE.

## 4. Materials and Methods

### 4.1. Study Population

The study included 23 women with RA, 22 with SLE, and 27 HCs, aged 18–55 years with a BMI of 18.5 to <30. Patients were recruited from Hospital Universitario San Ignacio and Hospital Militar Central (Bogotá, Colombia), with a disease duration of ≥2 years. RA patients met the ACR/EULAR 2010 criteria [47], with DAS28-ESR scores [48] of 3.2–5.2, while SLE patients met the SLICC 2012 criteria [49], with SLEDAI scores [50] of 4–12. Exclusion criteria included active smoking, obesity, pregnancy, systemic diseases (e.g., diabetes, cancer), antiphospholipid syndrome, systemic sclerosis, neuropsychiatric SLE, stage IV/V lupus nephritis, recent antibiotic or biologic therapy (in the last 3 months), and rituximab in the past year. HCs were matched by age and BMI, excluding those with a family history of autoimmunity and considering the above-listed criteria. Informed consent was obtained, and the study was approved by institutional ethics committees, adhering to the Declaration of Helsinki.

Fasting blood samples were collected in EDTA vacutainer tubes (BD, Franklin Lakes, NJ, USA), centrifuged at 3000 rpm (Allegra X, Beckman Coulter, Brea, CA, USA) for 10 min, and plasma was stored at −80 °C for metabolomics and lipidomics analysis. All participants underwent complete blood count and ESR analysis, while lipid profile (HDL, LDL, total cholesterol), glycemia, HbA1c, and CRP were also measured. SLE patients were additionally assessed for serum anti-dsDNA, C3, and C4 levels.

### 4.2. Metabolite Extraction

For lipid extraction, 350 µL of cold methanol (−20 °C) and 350 µL of methyl tert-butyl ether (MTBE) were added to 100 µL of plasma, vortex-mixed for 5 min, and centrifuged at 13,000 rpm (20 °C) for 10 min. Metabolomics extraction involved adding 600 µL of cold methanol to 200 µL of plasma, vortex-mixing for 3 min, and incubating at −20 °C for 20 min to precipitate proteins. The mixture was then centrifuged at 13,000 rpm (4 °C) for 10 min, and 100 µL of the supernatant was transferred to vials.

### 4.3. Liquid Chromatography Coupled to Quadrupole Time-of-Flight Mass Spectrometry (LC-QTOF-MS)

Plasma extracts were analyzed using an HPLC 1260 Infinity II system coupled to a Q-TOF 6545 mass spectrometer (Agilent Technologies, Santa Clara, CA, USA). For metabolomics, 2 µL were injected into a ZORBAX Eclipse Plus C18 column (50 × 2.1 mm, 1.8 µm; Agilent, Santa Clara, CA, USA) at 60 °C, with an elution gradient of 0.1% formic acid in water (Phase A) and acetonitrile (Phase B) at 0.6 mL/min. For lipidomics, 1 µL was injected into a C18 column at 40 °C, with an elution gradient of 10 mM ammonium acetate in H_2_O/methanol (90:10) (Phase A) and acetonitrile/methanol/isopropyl alcohol (20:30:50) (Phase B) at 0.6 mL/min. Mass spectrometry was performed in electrospray ionization (ESI) mode, with full scan detection (100–1100 *m*/*z*) and reference masses for correction.

### 4.4. Gas Chromatography Coupled to Quadrupole Time-of-Flight Mass Spectrometry (GC-QTOF-MS)

Metabolomics extracts were dried in a speed vac (35 °C, 3 h) before derivatization. Methoximation was performed with 10 µL O-methoxyamine in pyridine (15 mg/mL), incubated for 16 h, followed by silylation with 20 µL BSTFA + 1% TMCS, incubated at 70 °C for 1 h. Samples were injected (2 µL) into an Agilent 7890B GC system coupled to a Q-TOF 7250 (Agilent, Santa Clara, CA, USA) using an HP-5MS column (30 m, 0.25 mm, 0.25 µm; Agilent, Santa Clara, CA, USA) and helium as carrier gas (0.7 mL/min). The injection port was at 250 °C (split ratio 30:1), and the oven temperature was increased from 60 °C to 325 °C at 10 °C/min. Detection was performed using electron ionization (EI, 70 eV) in full scan mode (50–600 *m*/*z*).

### 4.5. Quality Control Samples

QC samples were prepared by pooling 30 µL of each plasma sample (RA, SLE, and HC groups) and were injected periodically to monitor system stability. LC-QTOF-MS QC samples were injected ten times at the beginning and then every eight samples, while GC-QTOF-MS QC samples were injected six times initially and then every five samples thereafter. PCA in Figure S1 confirmed QC clustering, ensuring analytical reproducibility.

### 4.6. Metabolomics and Lipidomics Data Analysis

LC-QTOF-MS data were processed using Agilent MassHunter Profinder B.10.0 (Agilent, Santa Clara, CA, USA) for peak alignment, deconvolution, and integration, followed by manual inspection of the peaks to further remove background noise and to verify the correct integration of the molecular features.

For GC-QTOF-MS data processing, the software Agilent Unknowns Analysis B.10.0 (Agilent, Santa Clara, CA, USA) was used for signal deconvolution. It also allowed compound identification using a mass spectral and retention index library of small chemical compounds [51]. The alignment was carried out using Agilent MassHunter Mass Profiler Professional B.15.0 software, and feature integration was performed with MassHunter Quantitative Data Analysis B.10.0 software (Agilent, Santa Clara, CA, USA).

To correct systematic variation, data were normalized by applying a QC-based normalization method called systematic error removal using random forest (SERRF) [52]. Afterward, the data were filtered for presence so that molecular features present in at least 80% of the samples of one of the study groups were kept. Molecular features were also filtered for reproducibility, excluding those with a percent coefficient variation (%CV) in QC greater than 20% for LC-QTOF-MS and greater than 30% for GC-QTOF-MS.

Molecular features were log-transformed and Pareto-scaled, with OPLS-DA (SIMCA 16.0, Umetrics, Sartorius Stedim Data Analytics AB, Umeå, Sweden) used to visualize group differences (Figure S2). Features were selected based on VIP > 1 and *p*-value < 0.05 (Wilcoxon–Mann–Whitney test, FDR-corrected). Metabolite identification was performed using CEU Mass Mediator (http://ceumass.eps.uspceu.es, accessed on 14 April 2025), matching exact masses to METLIN, KEGG, HMDB, and LipidMaps (≤10 ppm mass error), considering isotopic patterns, adducts, and retention times. MS/MS spectral confirmation was performed using MS-DIAL 4.8 (https://systemsomicslab.github.io/compms/msdial/main.html, accessed on 14 April 2025), Lipid Annotator v10.0, and Agilent MassHunter (Agilent, Santa Clara, CA, USA), with manual verification when needed. Confidence levels followed Metabolomics Standards Initiative (MSI) guidelines [53].

### 4.7. Statistical Analysis

Numerical covariates (e.g., age, BMI) were reported as median (IQR), and differences between study groups (RA vs. HC, SLE vs. HC, and RA vs. SLE) were assessed using the Kruskal–Wallis test, followed by Dunn’s test for multiple comparisons. Categorical variables were expressed as numbers and percentages, with Fisher’s exact test used for *p*-values. The calculations of Dunn’s test were performed using the R package “FSA” v 0.95 [54], and for the other statistical analysis, the R package “KODAMA” v 3.0 was used [55].

Logistic regression was used to classify RA vs. HC based on metabolite data. A binomial generalized linear model (GLM) was applied, and the predicted probabilities were used to generate a receiver operating characteristic (ROC) curve (pROC package, v 1.18.5 [56]) to evaluate classification performance.

### 4.8. Metabolite Mapping

Polar metabolite and lipid SMILES codes were used as input for the R package MetChem (v 0.4 [57]), which classified metabolites into chemically similar clusters with the clusters.detection function. The allbranches function further divided compounds into modules by cutting the branches of their corresponding hierarchical clustering tree, and the weighted metabolite chemical similarity analysis (WMCSA) function summarized metabolite concentrations of the modules. These concentrations were compared across groups using the Kruskal–Wallis test and Dunn’s test with Bonferroni correction.

### 4.9. Correlation Network Analysis

Spearman’s rank correlation coefficient (rho) was calculated to assess relationships between metabolite levels, cytokine levels, and blood/clinical parameters (*p* < 0.05 considered significant). A distance matrix was built from correlations, and the Floyd–Warshall algorithm was used to determine the shortest paths. Multidimensional scaling reduced dimensionality and the KODAMA algorithm [58] visualized networks in two-dimensional space.

For network analysis, metabolites with FDR < 0.05 were selected for RA vs. HC and SLE vs. HC comparisons. The metabolite-cytokine network included cytokines significantly increased in RA and SLE [32]. Clinical and demographic variables included HDL, LDL, total cholesterol, ESR, lymphocyte/neutrophil counts, and disease duration. DAS28-ESR was included for the RA network and the SLEDAI score for the SLE network.

## 5. Conclusions

This study highlights significant lipid profile changes in RA and SLE, with decreased phospholipids and sphingolipids and increased glycerolipids. The findings emphasize the link between lipid metabolism and cardiovascular disease (CVD) risk, with PE O-42:5 showing protective potential. The negative correlation between PE O-42:5 and cytokines highlights the connection between oxidative stress, inflammation, and atherosclerosis. The combined metabolite analysis—PS O-40:3 and FA 18:0 for RA and PC O-28:0 and DG 37:7 for SLE—enhances diagnostic performance, distinguishing patients from healthy controls. These results underscore the critical role of lipid metabolism in RA and SLE inflammation and suggest that ‘omics’ technologies could lead to the identification of promising new biomarkers.

## Figures and Tables

**Table 1:** Clinical and demographic characteristics of rheumatoid arthritis (RA) patients, systemic lupus erythematosus (SLE) patients, and healthy controls (HCs).

Feature	HC	RA	SLE	*p*-Value
age, median [IQR] ^(c)^	40 [31–49.5]	43 [39.5–49]	35 [25–40.75]	0.042
BMI, median [IQR]	24.5 [22.3–26.5]	25.1 [23.1–26.7]	25.1 [23.4–26.7]	0.771
familiar antecedent of autoimmunity				0.000546
no, *n* (%)	27 (100.0)	14 (60.9)	17 (77.3)	
yes, *n* (%)	0 (0.0)	9 (39.1)	5 (22.7)	
diagnostic time, median [IQR]	-	4.5 [3.0–9.0]	5.8 [3.8–11.3]	0.22
SLEDAI score, median [IQR]			6 [4–6]	
DAS28-ESR score, median [IQR]		3.6 [3.4–3.8]		
renal involvement in SLE				1
no, *n* (%)	-	-	12 (54.5)	
yes, *n* (%)	-	-	10 (45.5)	
HDL, median [IQR] ^(b)^	52.5 [45–60.2]	48 [37.5–56.5]	45.5 [37.5–49.5]	0.017
LDL, median [IQR] ^(b)^	123.6 [100.9–145.9]	109 [86.1–120.1]	93.5 [72.9–116.9]	0.022
total cholesterol, median [IQR] ^(b)^	202.4 [179.5–237.5]	172.9 [156.5–209.1]	164.4 [144.6–189.3]	0.009
triglycerides, median [IQR]	106.1 [88.6–144.7]	129.4 [89.2–166.8]	122.7 [86.8–171.2]	0.773
glucose, median [IQR]	86 [83.2–91]	83 [76.5–92]	82.5 [76–92.7]	0.273
HbA1c, median [IQR]	5.15 [5–5.4]	5.2 [5–5.315]	5.2 [5–5.4]	0.982
hematocrit, median [IQR] ^(a)^	43.7 [41.7–44.7]	40.6 [38.9–43.2]	41.7 [38.4–43.7]	0.027
hemoglobin, median [IQR] ^(a)^	14.6 [14.1–15.1]	13.6 [12.8–14.5]	13.85 [12.7–14.6]	0.008
ESR, median [IQR] ^(a)^	7 [5.3–10]	13 [6.5–19.5]	9.5 [6–16.2]	0.040
platelets, median [IQR]	273,500 [233,400–314,175]	289,500 [255,150–381,550]	312,950 [272,250–326,025]	0.259
leukocytes, median [IQR]	5900 [5025–6450]	6800 [5450–7850]	5600 [4675–6275]	0.152
lymphocytes, median [IQR] ^(b)^	1900 [1800–2175]	1700 [1250–2000]	1450 [1125–1775]	0.010
monocytes, median [IQR]	400 [300–500]	500 [400–550]	400 [300–500]	0.164
neutrophils, median [IQR] ^(a)^	3300 [2650–3800]	4400 [3300–5000]	3700 [3100–4075]	0.016
CRP, median [IQR]	0.26 [0.1–0.4]	0.34 [0.12–1.17]	0.31 [0.16–0.7]	0.346
RF, median [IQR]		102 [32–138.75]		
ACPA, median [IQR]		327 [173.75–579]		
anti-dsDNA, *n*(%)			6 (28.6)	
C3, median [IQR]			97.1 [81–109]	
C4, median [IQR]			20.9 [11.8–24.1]	

(a) Dunn’s test had a significant *p*-value (*p* < 0.05) for the comparison of RA vs. HC. (b) Dunn’s test had a significant *p*-value (*p* < 0.05) for the comparison of SLE vs. HC. (c) Dunn’s test had a significant *p*-value (*p* < 0.05) for the comparison of RA vs. SLE.

## Data Availability

The raw data supporting the conclusions of this article will be made available by the authors on request.

## Supplementary Materials

The supporting information can be downloaded at: https://www.mdpi.com/article/10.3390/ijms26104527/s1.

## References

[1] Theofilopoulos A.N., Kono D.H., Baccala R. (2018). The Multiple Pathways to Autoimmunity. Nat. Immunol..

[2] Vasquez-Canizares N., Wahezi D., Putterman C., Einstein A. (2018). Diagnostic and Prognostic Tests in Systemic Lupus Erythematosus. Best. Pract. Res. Clin. Rheumatol..

[3] Smolen J.S., Aletaha D., Barton A., Burmester G.R., Emery P., Firestein G.S., Kavanaugh A., McInnes I.B., Solomon D.H., Strand V. (2018). Rheumatoid arthritis. Nat. Rev. Dis. Primers.

[4] Li J., Tang H., Shang Z., Chen R., Meng X., Cheng X., Song Z., Li S., Zhang R., Lv W. (2025). Identifying functional subtypes and common mechanisms of rheumatoid arthritis and systemic lupus erythematosus. Genes Dis..

[5] Miller F.W. (2023). The increasing prevalence of autoimmunity and autoimmune diseases: An urgent call to action for improved understanding, diagnosis, treatment, and prevention. Curr. Opin. Immunol..

[6] Anaya J.-M., Beltrán S. (2023). The autoimmune tautology revisited. J. Transl. Autoimmun..

[7] Ellis J.A., Kemp A.S., Ponsonby A.L. (2014). Gene-environment interaction in autoimmune disease. Expert Rev. Mol. Med..

[8] Arriens C., Mohan C. (2013). Systemic lupus erythematosus diagnostics in the ‘omics’ era. Int. J. Clin. Rheumatol..

[9] Anaya J.M., Restrepo-Jiménez P., Ramírez-Santana C. (2018). The autoimmune ecology: An update. Curr. Opin. Rheumatol..

[10] Johnson C.H., Ivanisevic J., Siuzdak G. (2016). Metabolomics: Beyond biomarkers and towards mechanisms. Nat. Rev. Mol. Cell Biol..

[11] Priori R., Scrivo R., Brandt J., Valerio M., Casadei L., Valesini G., Manetti C. (2013). Metabolomics in rheumatic diseases: The potential of an emerging methodology for improved patient diagnosis, prognosis, and treatment efficacy. Autoimmun. Rev..

[12] Wang A., Luan H.H., Medzhitov R. (2019). An evolutionary perspective on immunometabolism. Science.

[13] Huang N., Perl A. (2018). Metabolism as a Target for Modulation in Autoimmune Diseases. Trends Immunol..

[14] Weyand C.M., Goronzy J.J. (2017). Immunometabolism in early and late stages of rheumatoid arthritis. Nat. Rev. Rheumatol..

[15] Morel L. (2017). Immunometabolism in systemic lupus erythematosus. Nat. Rev. Rheumatol..

[16] Fernández-Ávila D.G., Rincón-Riaño D.N., Bernal-Macías S., Gutiérrez Dávila J.M., Rosselli D. (2019). Prevalencia de la artritis reumatoide en Colombia según información del Sistema Integral de Información de la Protección Social. Rev. Colomb. Reumatol..

[17] Fernández-Ávila D.G., Bernal-Macías S., Rincón-Riaño D.N., Gutiérrez Dávila J.M., Rosselli D. (2019). Prevalence of systemic lupus erythematosus in Colombia: Data from the national health registry 2012–2016. Lupus.

[18] Londoño J., Ballestas I.P., Cuervo F., Angarita I., Giraldo R., Rueda J.C., Ballesteros J.G., Baquero R., Forero E., Cardiel M. (2018). Prevalencia de la enfermedad reumática en Colombia, según estrategia COPCORD-Asociación Colombiana de Reumatología. Estudio de prevalencia de enfermedad reumática en población colombiana mayor de 18 años. Rev. Colomb. De Reumatol..

[19] Sahin D., Di Matteo A., Emery P. (2024). Biomarkers in the diagnosis, prognosis and management of rheumatoid arthritis: A comprehensive review. Ann. Clin. Biochem. Int. J. Lab. Med..

[20] Sandhu V., Quan M. (2017). SLE and Serum Complement: Causative, Concomitant or Coincidental?. Open Rheumatol. J..

[21] Arriens C., Wren J.D., Munroe M.E., Mohan C. (2017). Systemic lupus erythematosus biomarkers: The challenging quest. Rheumatology.

[22] Guma M., Tiziani S., Firestein G.S. (2016). Metabolomics in rheumatic diseases: Desperately seeking biomarkers. Nat. Rev. Rheumatol..

[23] Duarte-Delgado N.P., Cala M.P., Barreto A., Rodríguez C.L.S. (2022). Metabolites and metabolic pathways associated with rheumatoid arthritis and systemic lupus erythematosus. J. Transl. Autoimmun..

[24] Li S., Todor A., Luo R. (2016). Blood transcriptomics and metabolomics for personalized medicine. Comput. Struct. Biotechnol. J..

[25] Szabó M.Z., Szodoray P., Kiss E. (2017). Dyslipidemia in systemic lupus erythematosus. Immunol. Res..

[26] Yan J., Yang S., Han L., Ba X., Shen P., Lin W., Li T., Zhang R., Huang Y., Huang Y. (2023). Dyslipidemia in rheumatoid arthritis: The possible mechanisms. Front. Immunol..

[27] Cui W., Liu D., Gu W., Chu B. (2021). Peroxisome-driven ether-linked phospholipids biosynthesis is essential for ferroptosis. Cell Death Differ..

[28] Codini M., Garcia-Gil M., Albi E. (2021). Cholesterol and sphingolipid enriched lipid rafts as therapeutic targets in cancer. Int. J. Mol. Sci..

[29] Harden O.C., Hammad S.M. (2020). Sphingolipids and Diagnosis, Prognosis, and Organ Damage in Systemic Lupus Erythematosus. Front. Immunol..

[30] Zhang B.H., Yin F., Qiao Y.N., Guo S.D. (2022). Triglyceride and Triglyceride-Rich Lipoproteins in Atherosclerosis. Front. Mol. Biosci..

[31] Kolczynska K., Loza-Valdes A., Hawro I., Sumara G. (2020). Diacylglycerol-evoked activation of PKC and PKD isoforms in regulation of glucose and lipid metabolism: A review. Lipids Health Dis..

[32] Duarte-Delgado N.P., Segura K., Gómez O., Pulido S., Tovar-Sánchez C., Bello-Gualtero J.M., Fernández-Ávila D.G., Amado-Garzón S.B., Romero-Sanchez C., Cacciatore S. (2024). Cytokine profiles and their correlation with clinical and blood parameters in rheumatoid arthritis and systemic lupus erythematosus. Sci. Rep..

[33] Zhou B., Xia Y., She J. (2020). Dysregulated serum lipid profile and its correlation to disease activity in young female adults diagnosed with systemic lupus erythematosus: A cross-sectional study. Lipids Health Dis..

[34] Rodríguez-Carrio J., Alperi-López M., López P., López-Mejías R., Alonso-Castro S., Abal F., Ballina-García F.J., González-Gay M.Á., Suárez A. (2017). High triglycerides and low high-density lipoprotein cholesterol lipid profile in rheumatoid arthritis: A potential link among inflammation, oxidative status, and dysfunctional high-density lipoprotein. J. Clin. Lipidol..

[35] Cas M.D., Roda G., Li F., Secundo F. (2020). Functional lipids in autoimmune inflammatory diseases. Int. J. Mol. Sci..

[36] Wang R., Li B., Lam S.M., Shui G. (2020). Integration of lipidomics and metabolomics for in-depth understanding of cellular mechanism and disease progression. J. Genet. Genom..

[37] Fang L., Mundra P.A., Fan F., Galvin A., Weir J.M., Wong G., Chin-Dusting J., Cicuttini F., Meikle P., Dart A.M. (2016). Plasma lipidomic profiling in patients with rheumatoid arthritis. Metabolomics.

[38] Hu C., Zhou J., Yang S., Li H., Wang C., Fang X., Fan Y., Zhang J., Han X., Wen C. (2016). Oxidative stress leads to reduction of plasmalogen serving as a novel biomarker for systemic lupus erythematosus. Free Radic. Biol. Med..

[39] Robinson G., Pineda-Torra I., Ciurtin C., Jury E.C. (2022). Lipid metabolism in autoimmune rheumatic disease: Implications for modern and conventional therapies. J. Clin. Investig..

[40] Lei Q., Yang J., Li L., Zhao N., Lu C., Lu A., He X. (2023). Lipid metabolism and rheumatoid arthritis. Front. Immunol..

[41] Wójcik P., Gęgotek A., Žarković N., Skrzydlewska E. (2021). Oxidative stress and lipid mediators modulate immune cell functions in autoimmune diseases. Int. J. Mol. Sci..

[42] Frommer K.W., Schäffler A., Rehart S., Lehr A., Müller-Ladner U., Neumann E. (2015). Free fatty acids: Potential proinflammatory mediators in rheumatic diseases. Ann. Rheum. Dis..

[43] Masuoka S., Nishio J., Yamada S., Saito K., Kaneko K., Kaburaki M., Tanaka N., Sato H., Muraoka S., Kawazoe M. (2024). Relationship Between the Lipidome Profile and Disease Activity in Patients with Rheumatoid Arthritis. Inflammation.

[44] Johri N., Varshney S., Gandha S., Maurya A., Mittal P., Jangra S., Garg R., Saraf A. (2023). Association of cardiovascular risks in rheumatoid arthritis patients: Management, treatment and future perspectives. Health Sci. Rev..

[45] Ferreira H.B., Melo T., Paiva A., Domingues M.D.R. (2021). Insights in the role of lipids, oxidative stress and inflammation in rheumatoid arthritis unveiled by new trends in lipidomic investigations. Antioxidants.

[46] Sutter I., Velagapudi S., Othman A., Riwanto M., Manz J., Rohrer L., Rentsch K., Hornemann T., Landmesser U., von Eckardstein A. (2015). Plasmalogens of high-density lipoproteins (HDL) are associated with coronary artery disease and anti-apoptotic activity of HDL. Atherosclerosis.

[47] Kay J., Upchurch K.S. (2012). ACR/EULAR 2010 rheumatoid arthritis classification criteria. Rheumatology.

[48] Prevoo M.L.L., Van’T Hof M.A., Kuper H.H., Van Leeuwen M.A., Van De Putte L.B.A., Van Riel P.L.C.M. (1995). Modified disease activity scores that include twenty-eight-joint counts development and validation in a prospective longitudinal study of patients with rheumatoid arthritis. Arthritis Rheum..

[49] Petri M., Orbai A.M., Alarcón G.S., Gordon C., Merrill J.T., Fortin P.R., Bruce I.N., Isenberg D., Wallace D.J., Nived O. (2012). Derivation and Validation of Systemic Lupus International Collaborating Clinics Classification Criteria for Systemic Lupus Erythematosus. Arthritis Rheum..

[50] Bombardier C., Gladman D.D., Urowitz M.B., Caron D., Chang C.H. (1992). Derivation of the SLEDAI. A disease activity index for lupus patients. Comm. Progn. Stud. SLE. Arthritis Rheum..

[51] Kind T., Wohlgemuth G., Lee D.Y., Lu Y., Palazoglu M., Shahbaz S., Fiehn O. (2009). FiehnLib: Mass spectral and retention index libraries for metabolomics based on quadrupole and time-of-flight gas chromatography/mass spectrometry. Anal. Chem..

[52] Fan S., Kind T., Cajka T., Hazen S.L., Tang W.W., Kaddurah-Daouk R., Irvin M.R., Arnett D.K., Barupal D.K., Fiehn O. (2019). Systematic Error Removal Using Random Forest for Normalizing Large-Scale Untargeted Lipidomics Data. Anal. Chem..

[53] Schymanski E.L., Jeon J., Gulde R., Fenner K., Ruff M., Singer H.P., Hollender J. (2014). Identifying small molecules via high resolution mass spectrometry: Communicating confidence. Environ. Sci. Technol..

[54] Ogle D., Doll J., Wheeler A., Dinno A. *FSA: Simple Fisheries Stock Assessment Methods*, R package version 0.9.5, 2023. https://CRAN.R-project.org/package=FSA.

[55] Cacciatore S., Tenori L., Luchinat C., Bennett P.R., MacIntyre D.A. (2017). KODAMA: An R package for knowledge discovery and data mining. Bioinformatics.

[56] Robin X., Turck N., Hainard A., Tiberti N., Lisacek F., Sanchez J.C., Müller M. (2011). pROC: An open-source package for R and S+ to analyze and compare ROC curves. BMC Bioinform..

[57] Abdel-Shafy E.A., Melak T., MacIntyre D.A., Zadra G., Zerbini L.F., Piazza S., Cacciatore S. (2023). MetChem: A new pipeline to explore structural similarity across metabolite modules. Bioinform. Adv..

[58] Cacciatore S., Luchinat C., Tenori L. (2014). Knowledge discovery by accuracy maximization. Proc. Natl. Acad. Sci. USA.

