# Supplementary material for: Metabolic Alterations in Colombian Women with Rheumatoid Arthritis and Systemic Lupus Erythematosus Reveal Potential Lipid Biomarkers Associated with Inflammation and Cardiovascular Risk
Source: Int J Mol Sci. 2025 May 9;26(10):4527. doi: 10.3390/ijms26104527 (PMC12111616; doi:10.3390/ijms26104527)
Supplement: Supplementary file 1 [file ijms-26-04527-s001.zip › supplementary figures.pdf]

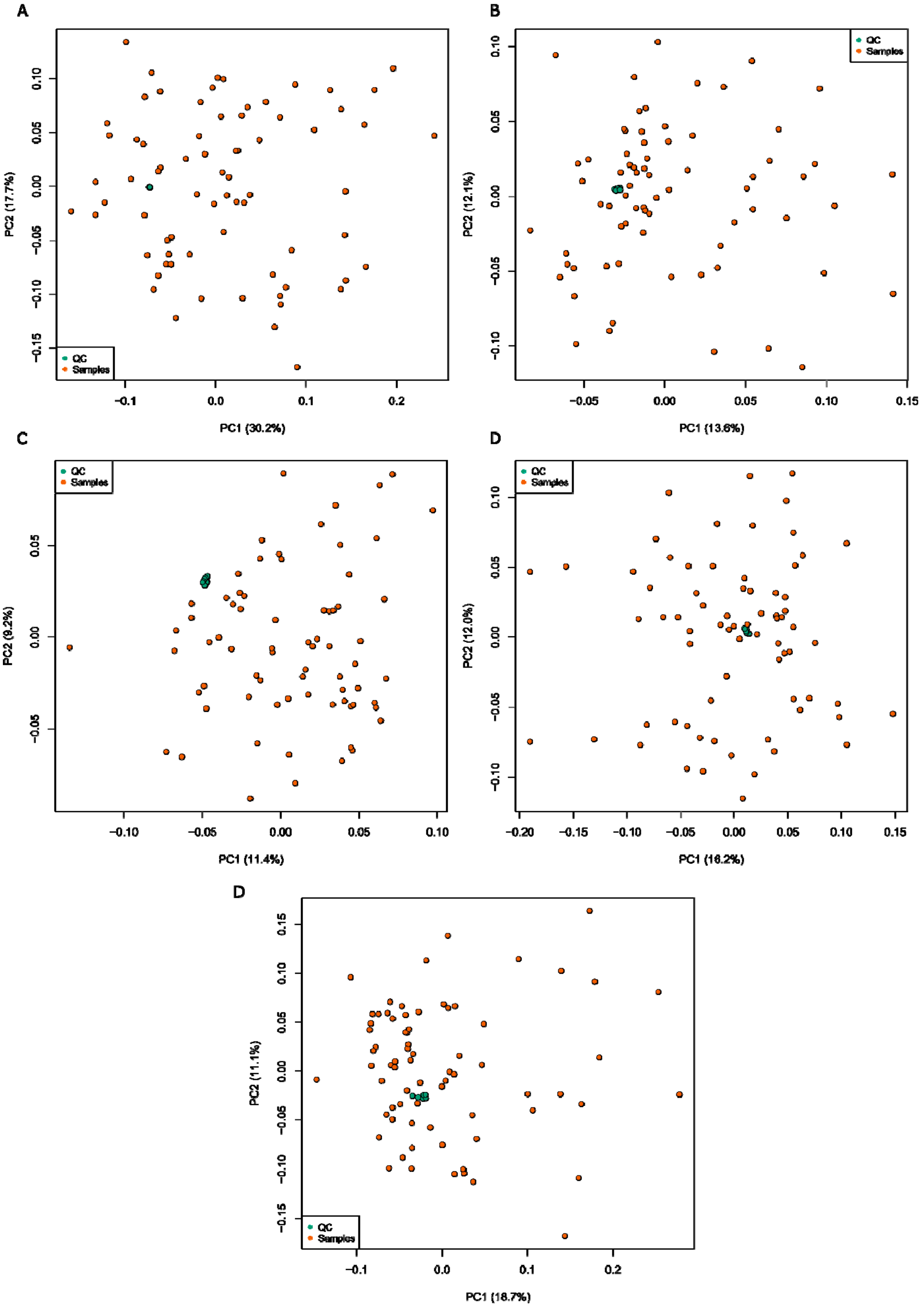

Figure S1. PCA analysis showing the clustering of the QC samples. A) Lipidomics ESI+. B) Lipidomics ESI-. C) Metabolomics ESI+. D) Metabolomics ESI-. E) GC-MS

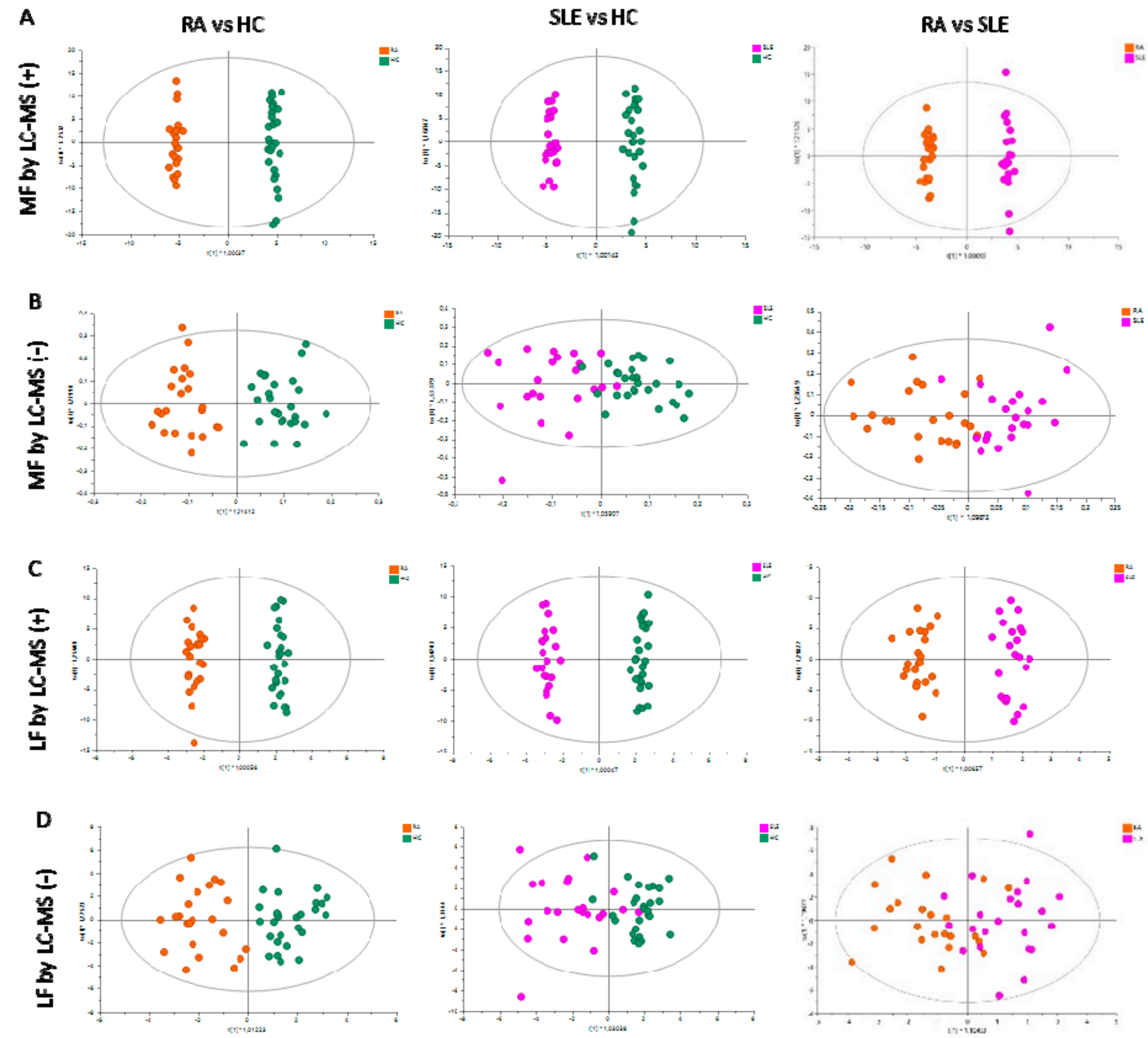

Figure S2. Multivariate supervised analysis. The results of the OPLS-DA analysis are shown for the comparisons (SLE vs HC, RA vs HC and RA vs SLE), including the values for R^2 (explained variance by the model), Q^2 (predicted variance by the model) and CV-ANOVA(cross-validated predictive residuals). Each colored point represents a patient with RA (orange), SLE (pink) or HC (green). **A)** Metabolomics by LC-MS (+) left RA VS HC R^2X(cum): 0.376, R^2Y(cum): 0.996, Q^2(cum): 0.915 CV-ANOVA: 9.78874e-18, middle SLE vs HC R^2X(cum): 0.379, R^2Y(cum): 0.988, Q^2(cum): 0.788 CV-ANOVA: 5.67971e-10, right RA vs SLE R^2X(cum): 0.332, R^2Y(cum): 0.994, Q^2(cum): 0.782 CV-ANOVA: 1.95439e-08 **B)** Metabolomics by LC-MS (-). Left RA VS HC R^2X(cum): 0.29, R^2Y(cum): 0.874, Q^2(cum): 0.607 CV-ANOVA: 2.13603e-07, middle SLE vs HC R^2X(cum): 0.224, R^2Y(cum): 0.659, Q^2(cum): 0.265 CV-ANOVA: 0.0071633, right RA vs SLE R^2X(cum): 0.216, R^2Y(cum): 0.616, Q^2(cum): 0.191 CV-ANOVA: 0.0896846 **C)** Lipidomics by LC-MS (+) left RA VS HC R^2X(cum): 0.739, R^2Y(cum): 0.984, Q^2(cum): 0.685 CV-ANOVA: 0.000519269, middle SLE vs HC R^2X(cum): 0.751, R^2Y(cum): 0.989, Q^2(cum): 0.592 CV-ANOVA: 0.00477725, right RA vs SLE R^2X(cum): 0.332, R^2Y(cum): 0.994, Q^2(cum): 0.782 CV-ANOVA: 1.95439e-08 **D)** Lipidomics by LC-MS (-) left RA VS HC R^2X(cum): 0.313, R^2Y(cum): 0.806, Q^2(cum): 0.465 CV-ANOVA: 9.11431e-05, middle SLE vs HC R^2X(cum): 0.295, R^2Y(cum): 0.626, Q^2(cum): 0.302 CV-ANOVA: 0.00281169, right RA vs SLE $R^2X_{(cum):}$ 0.239, $R^2Y_{(cum):}$ 0.515, $Q^2_{(cum)}$: -0.0895 CV-ANOVA: 0.99465

Figure S3. Heatmaps for the metabolites of each of the six clusters

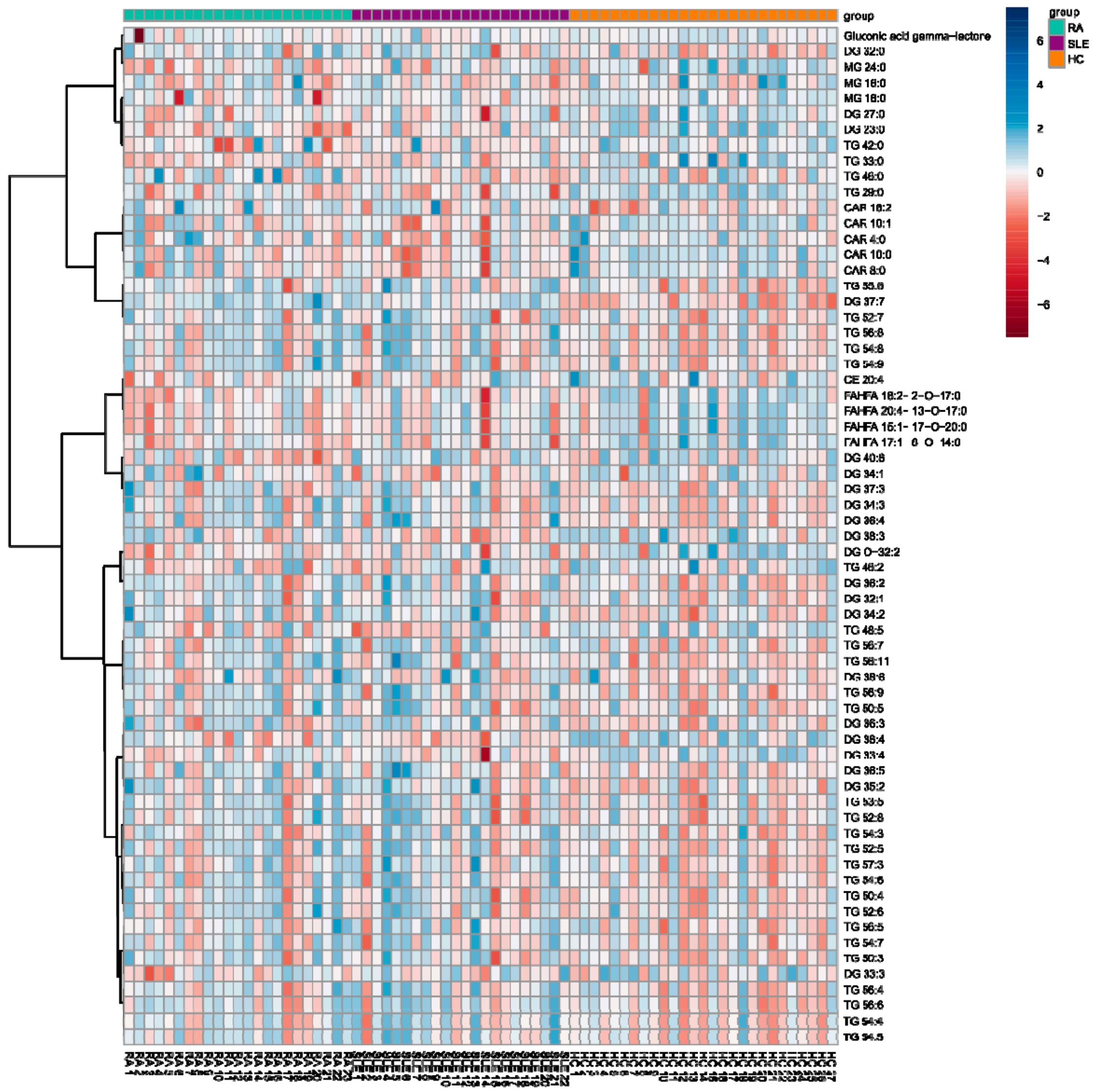

Figure S3a. Heatmap for metabolites of cluster 1.

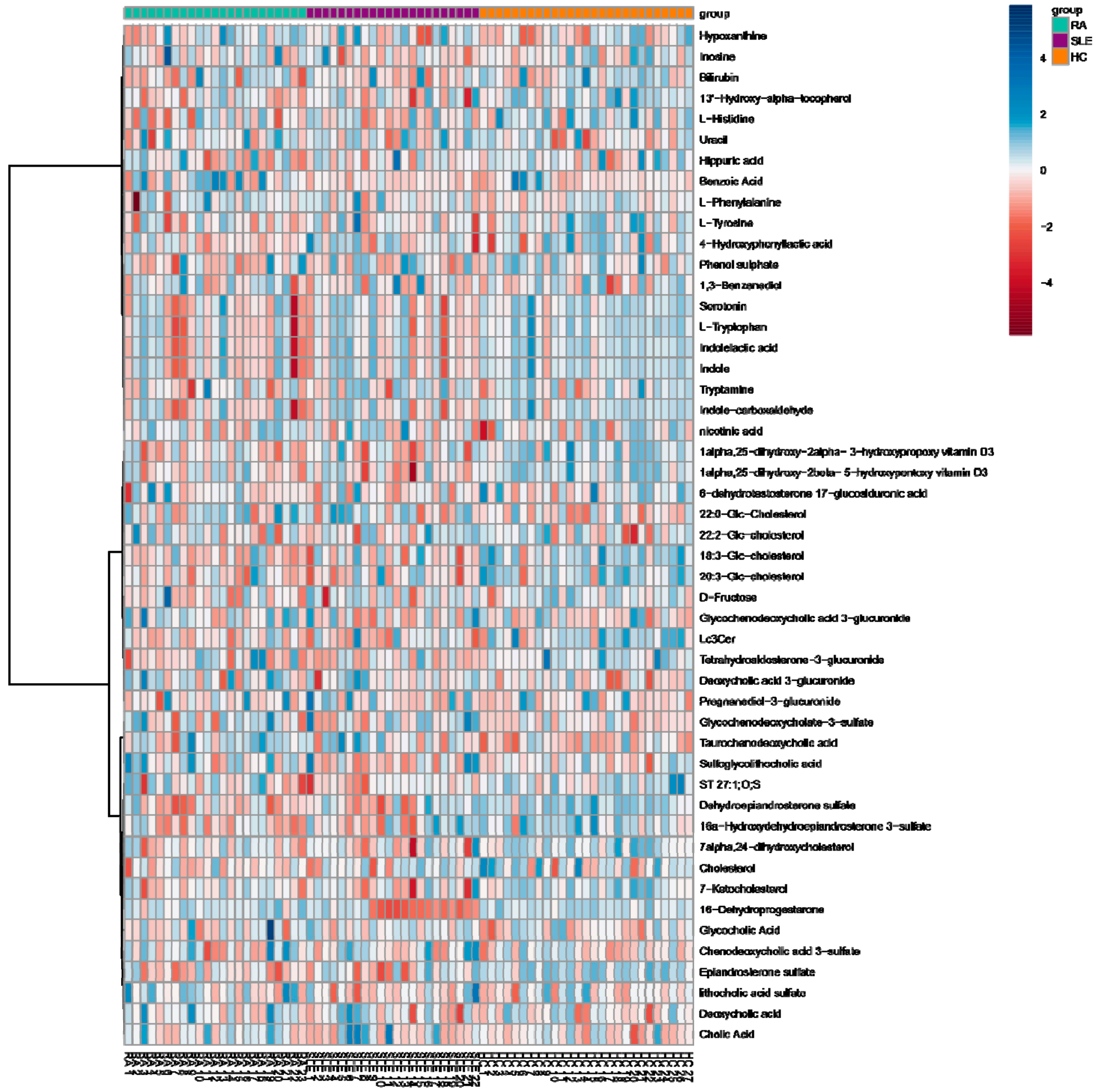

Figure S3b. Heatmap for metabolites of cluster 2.

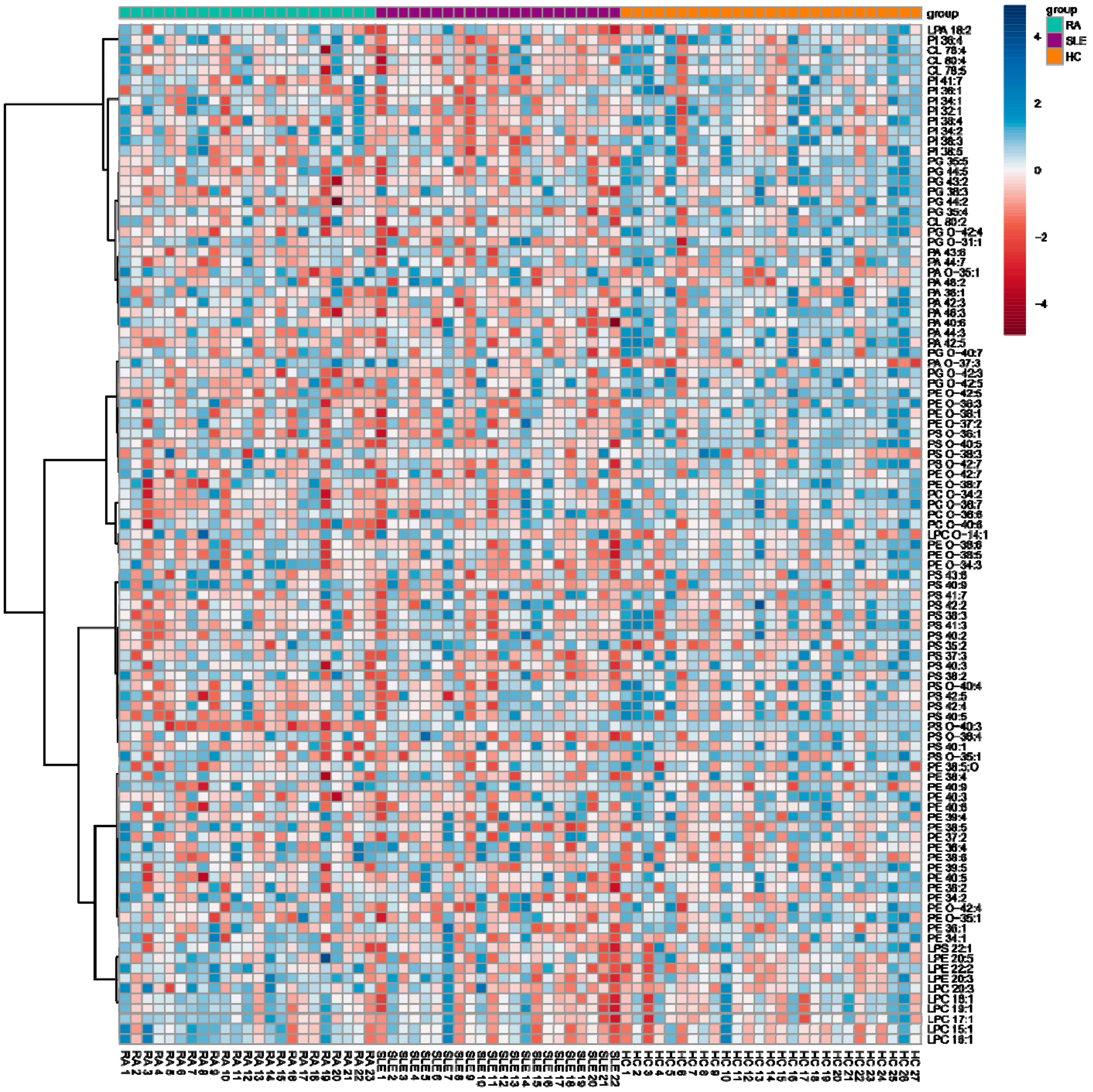

Figure S3c. Heatmap for metabolites of cluster 3.

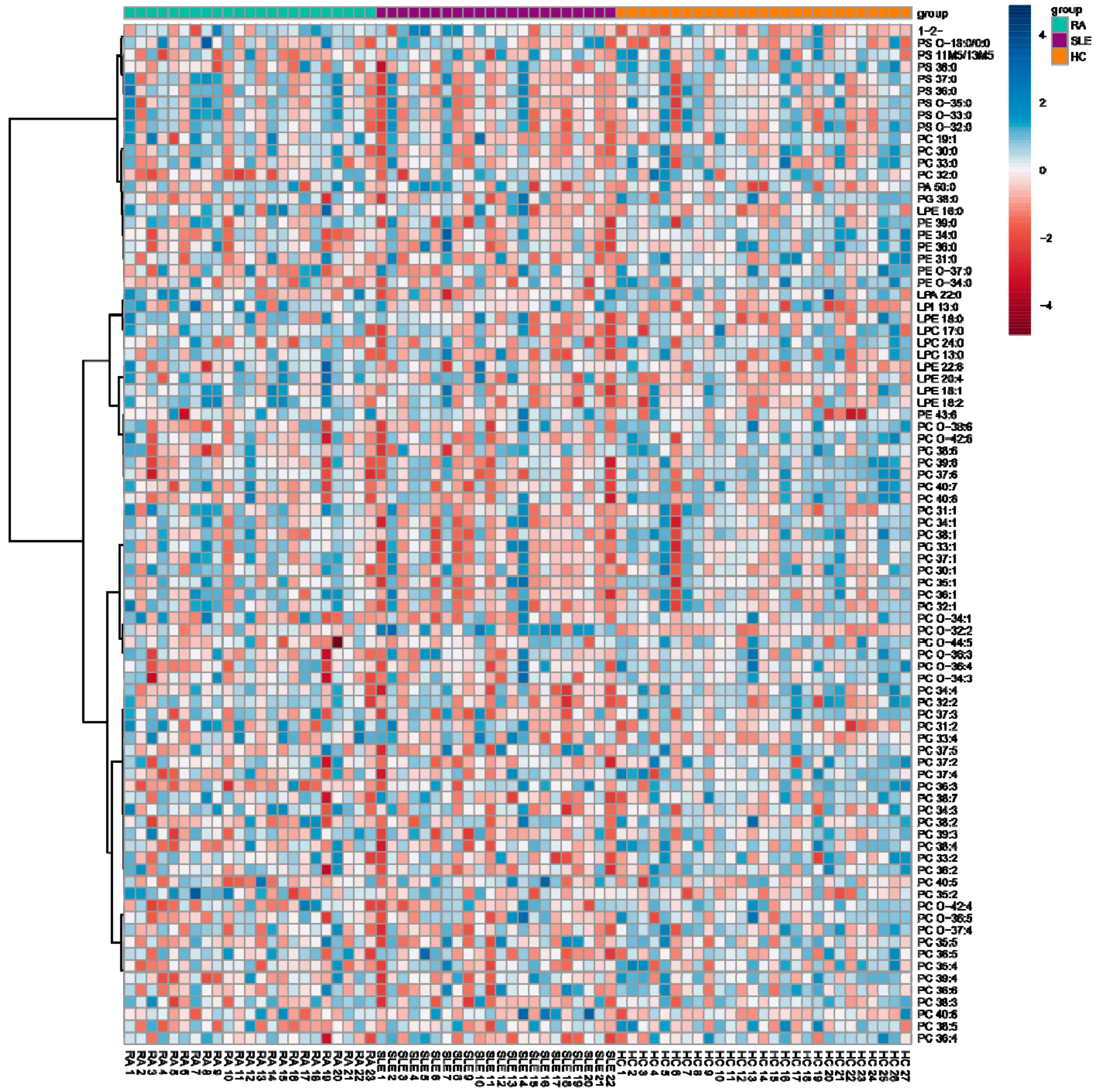

Figure S3d. Heatmap for metabolites of cluster 4.

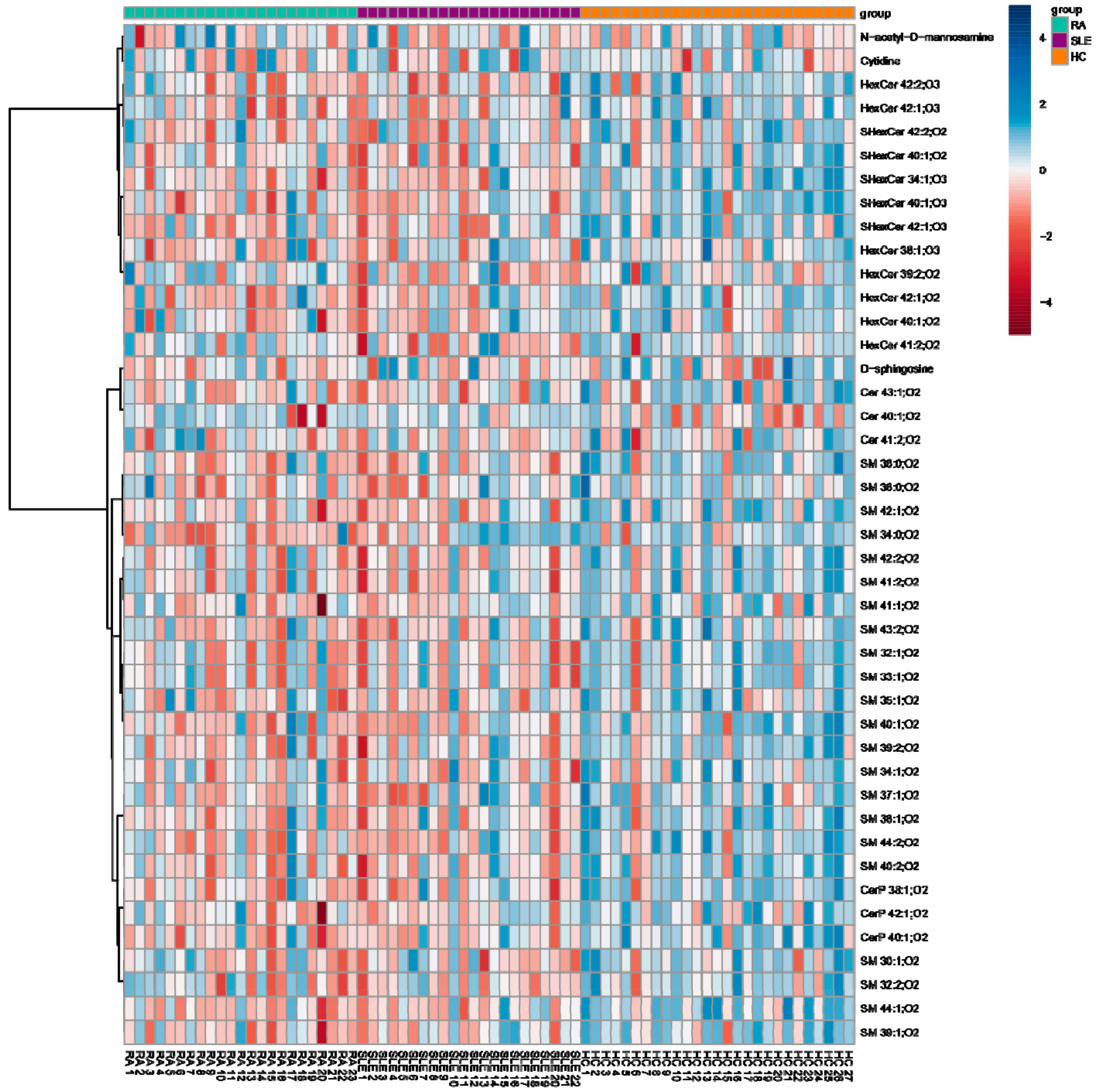

Figure S3e. Heatmap for metabolites of cluster 5.

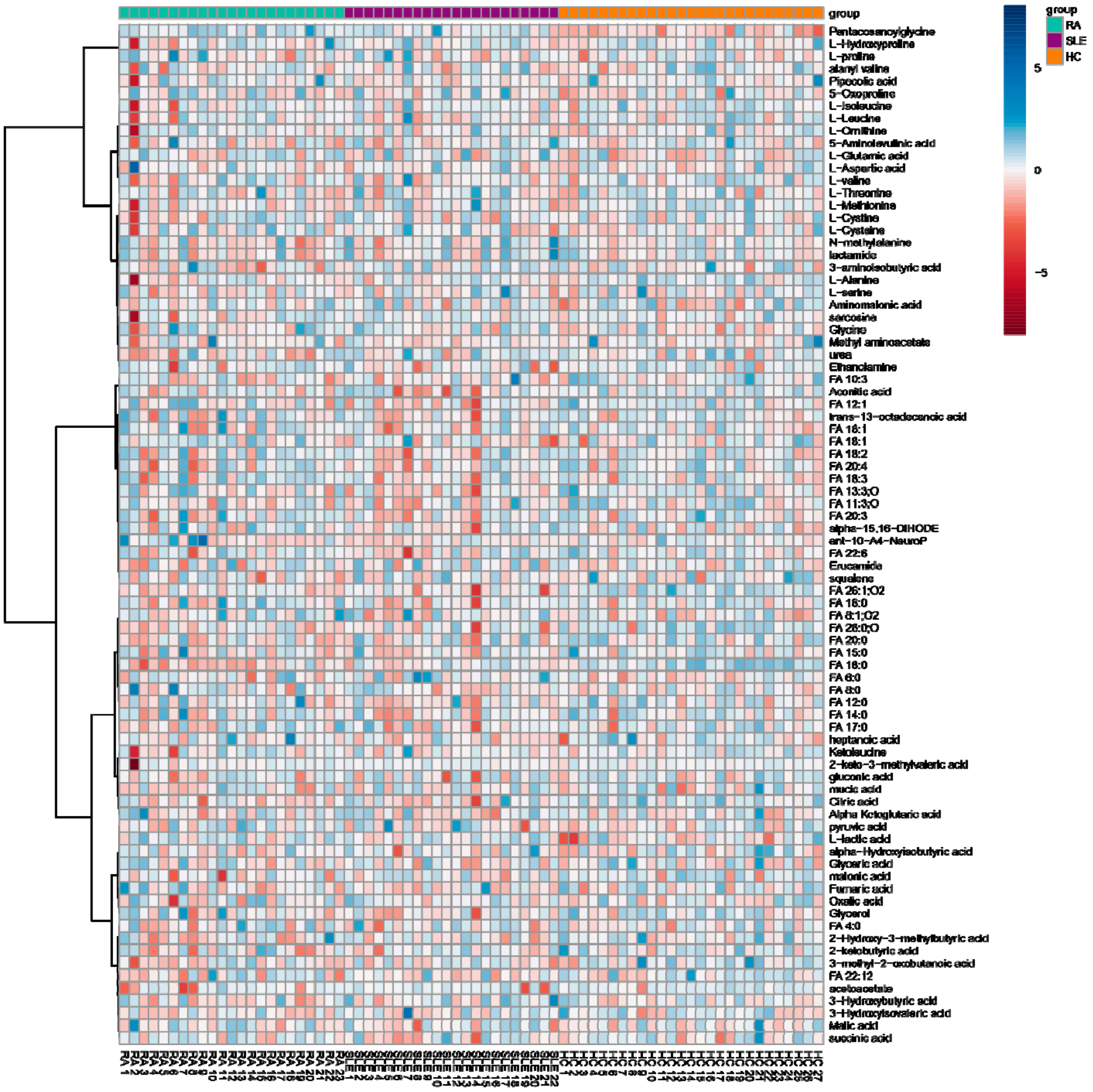

Figure S3f. Heatmap for metabolites of cluster 6.

Figure S4. Heatmaps of Modules Composing Each Cluster with Dunn’s Test Significance Values for Multiple Comparisons

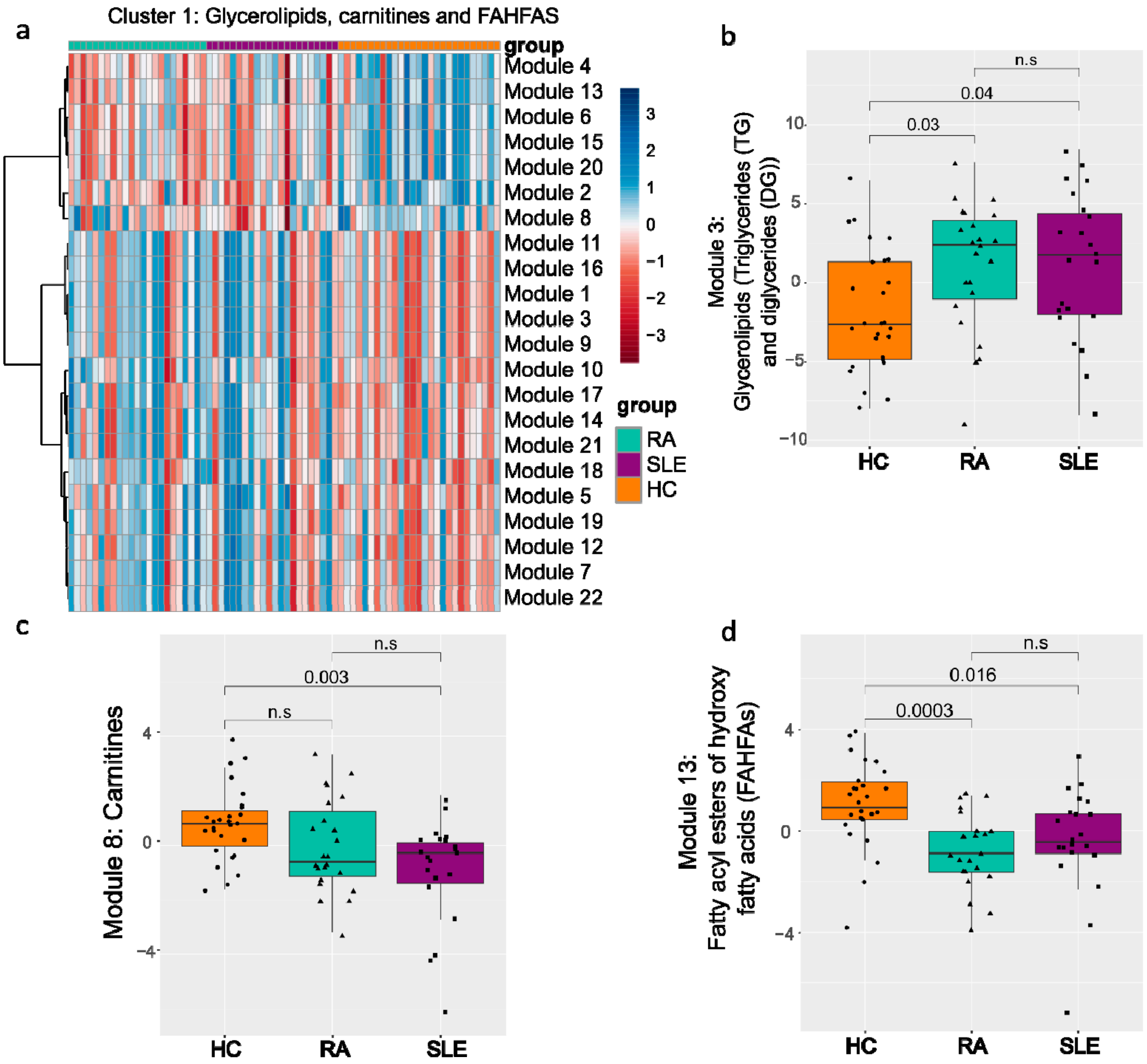

Figure S4a

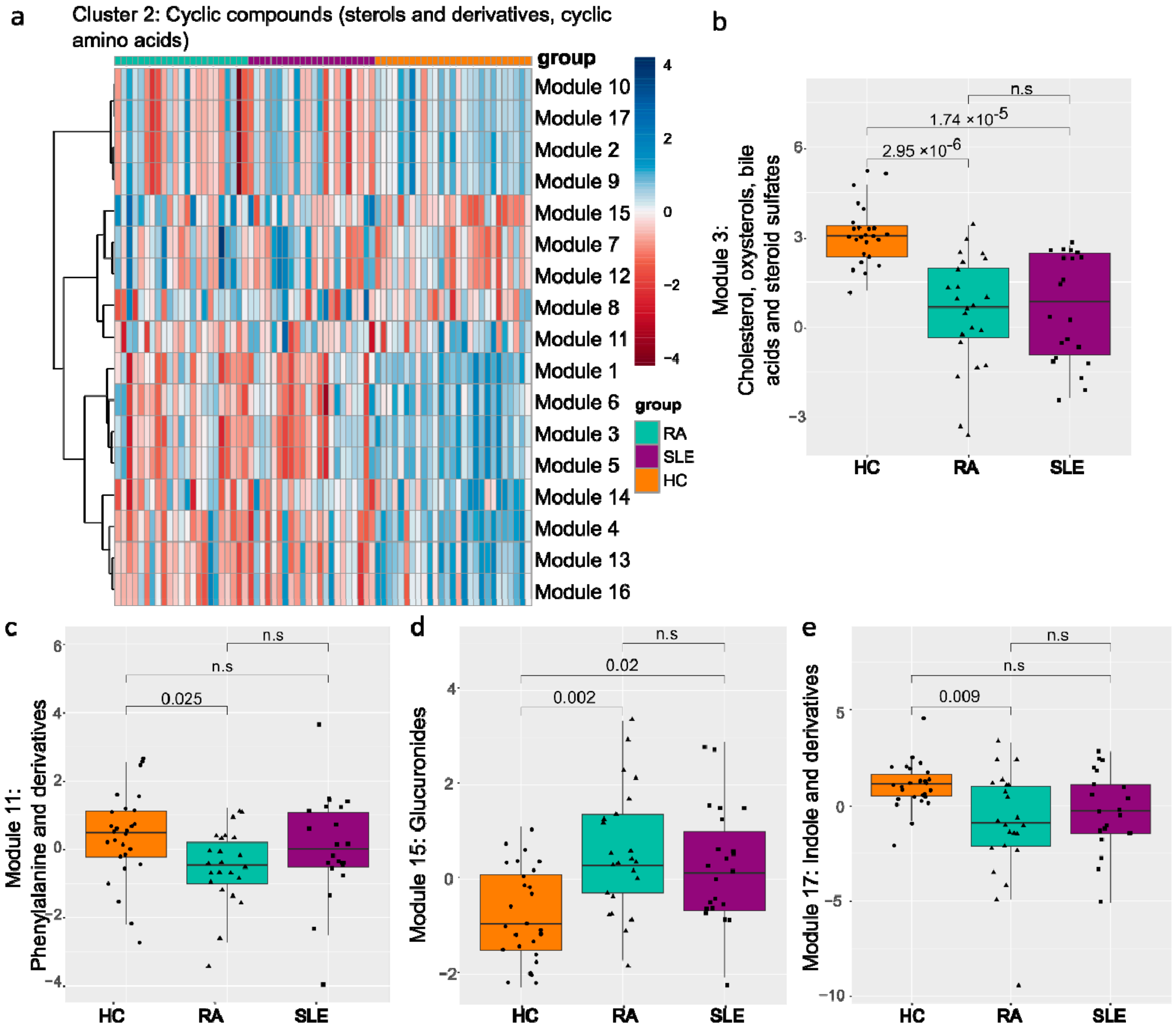
a
Cluster 2: Cyclic compounds (sterols and derivatives, cyclic amino acids)
group
Module 10
Module 17
Module 2
Module 9
Module 15
Module 7
Module 12
Module 8
Module 11
Module 1
Module 6
Module 3
Module 5
Module 14
Module 4
Module 13
Module 16
4
2
0
−2
−4
group
RA
SLE
HC
b
Module 3: Cholesterol, oxysterols, bile acids and steroid sulfates
n.s
1.74 ×10-5
2.95 ×10-6
HC
RA
SLE
c
Module 11: Phenylalanine and derivatives
n.s
n.s
0.025
HC
RA
SLE
d
Module 15: Glucuronides
n.s
0.02
0.002
HC
RA
SLE
e
Module 17: Indole and derivatives
n.s
n.s
0.009
HC
RA
SLE

Figure S4b

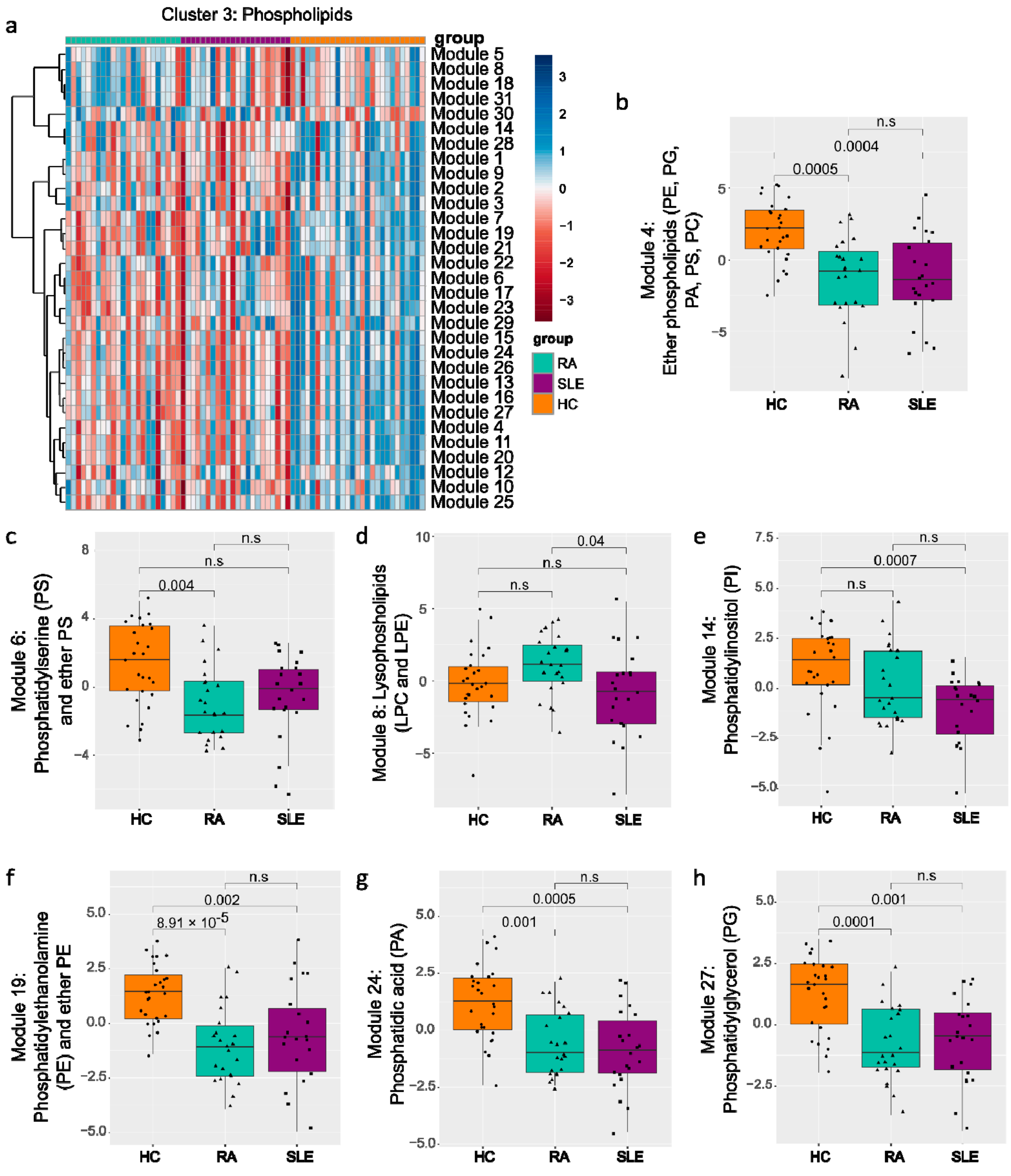

a
Cluster 3: Phospholipids
group
Module 5
Module 8
Module 18
Module 31
Module 30
Module 14
Module 28
Module 1
Module 9
Module 2
Module 3
Module 7
Module 19
Module 21
Module 22
Module 6
Module 17
Module 23
Module 29
Module 15
Module 24
Module 26
Module 13
Module 16
Module 27
Module 4
Module 11
Module 20
Module 12
Module 10
Module 25
3
2
1
0
−1
−2
−3
group
RA
SLE
HC
b
Module 4:
Ether phospholipids (PE, PG, PA, PS, PC)
n.s
0.0004
0.0005
HC
RA
SLE
c
Module 6:
Phosphatidylserine (PS) and ether PS
n.s
n.s
0.004
HC
RA
SLE
d
Module 8: Lysophosholipids (LPC and LPE)
0.04
n.s
n.s
HC
RA
SLE
e
Module 14:
Phosphatidylinositol (PI)
n.s
0.0007
n.s
HC
RA
SLE
f
Module 19:
Phosphatidylethanolamine (PE) and ether PE
n.s
0.002
8.91 × 10−5
HC
RA
SLE
g
Module 24:
Phosphatidic acid (PA)
n.s
0.0005
0.001
HC
RA
SLE
h
Module 27:
Phosphatidylglycerol (PG)
n.s
0.001
0.0001
HC
RA
SLE

Figure S4c

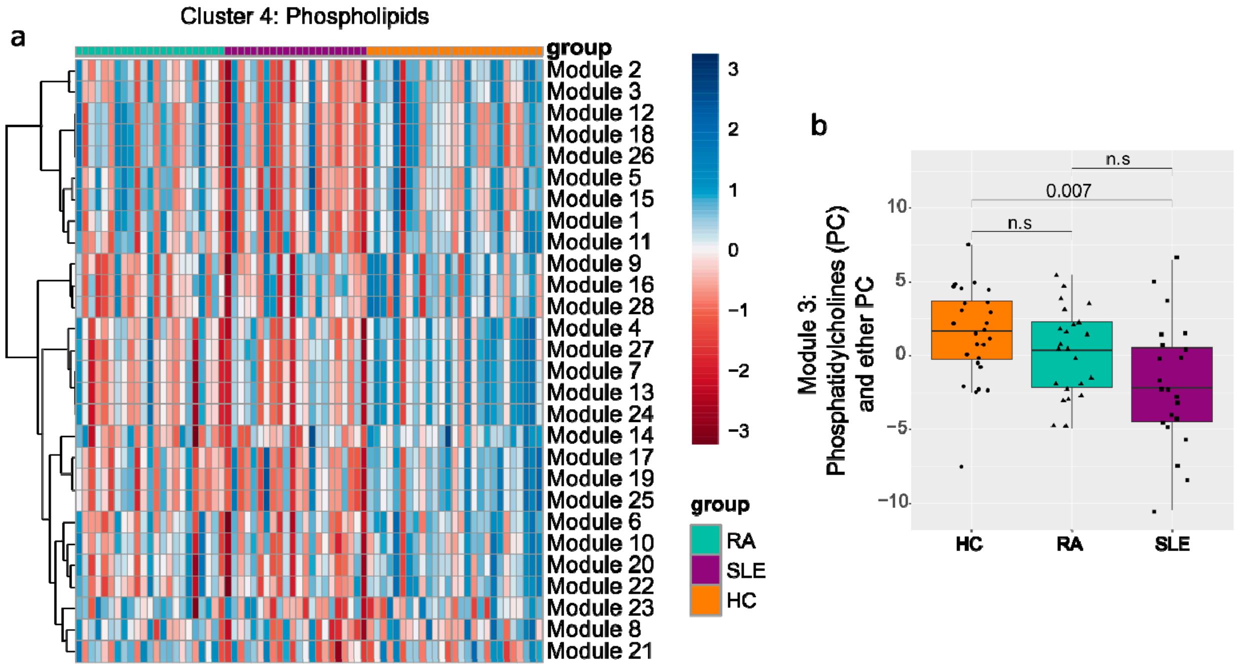
a
Cluster 4: Phospholipids
group
Module 2
Module 3
Module 12
Module 18
Module 26
Module 5
Module 15
Module 1
Module 11
Module 9
Module 16
Module 28
Module 4
Module 27
Module 7
Module 13
Module 24
Module 14
Module 17
Module 19
Module 25
Module 6
Module 10
Module 20
Module 22
Module 23
Module 8
Module 21
3
2
1
0
−1
−2
−3
group
RA
SLE
HC
b
Module 3:
Phosphatidylcholines (PC)
and ether PC
n.s
0.007
n.s
10
5
0
−5
−10
HC
RA
SLE

Figure S4d

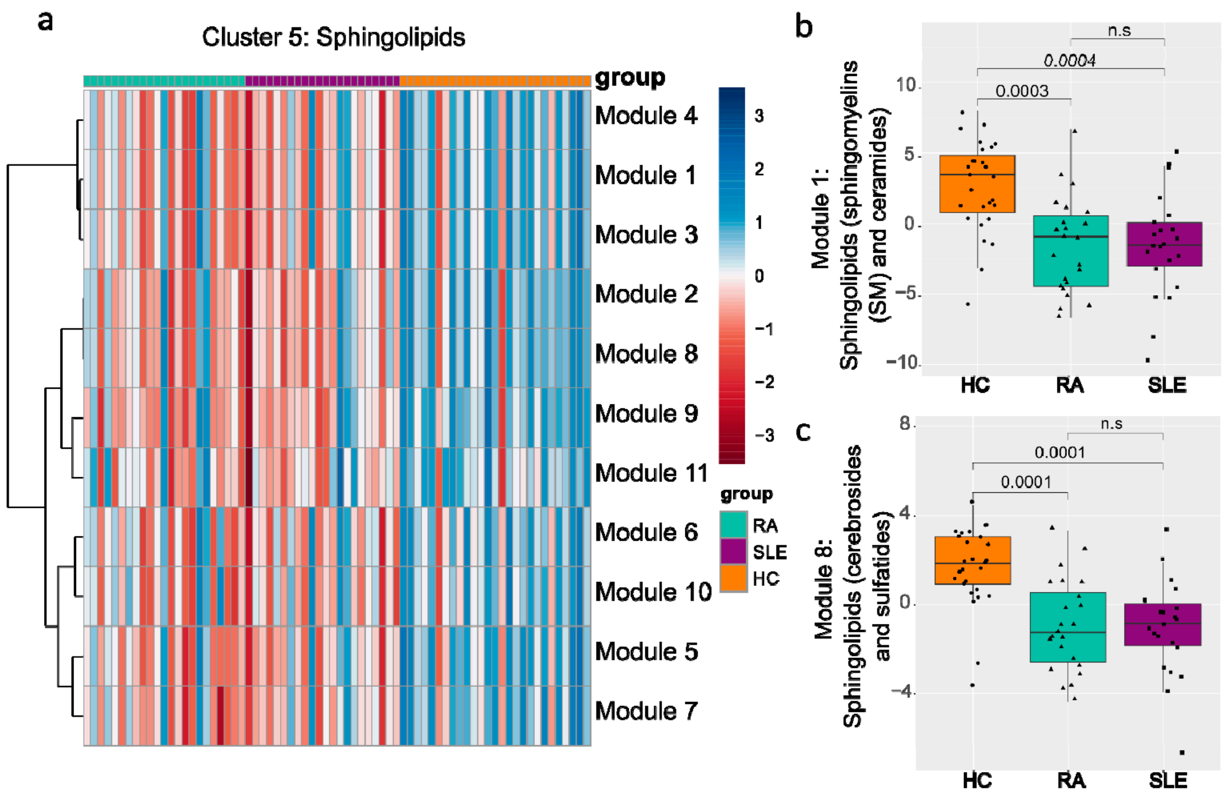

a
Cluster 5: Sphingolipids
group
Module 4
Module 1
Module 3
Module 2
Module 8
Module 9
Module 11
Module 6
Module 10
Module 5
Module 7
3
2
1
0
−1
−2
−3
group
RA
SLE
HC
b
Module 1: Sphingolipids (sphingomyelins (SM) and ceramides)
n.s
0.0004
0.0003
10
5
0
−5
−10
HC
RA
SLE
c
Module 8: Sphingolipids (cerebrosides and sulfatides)
n.s
0.0001
0.0001
8
4
0
−4
HC
RA
SLE

Figure S4e

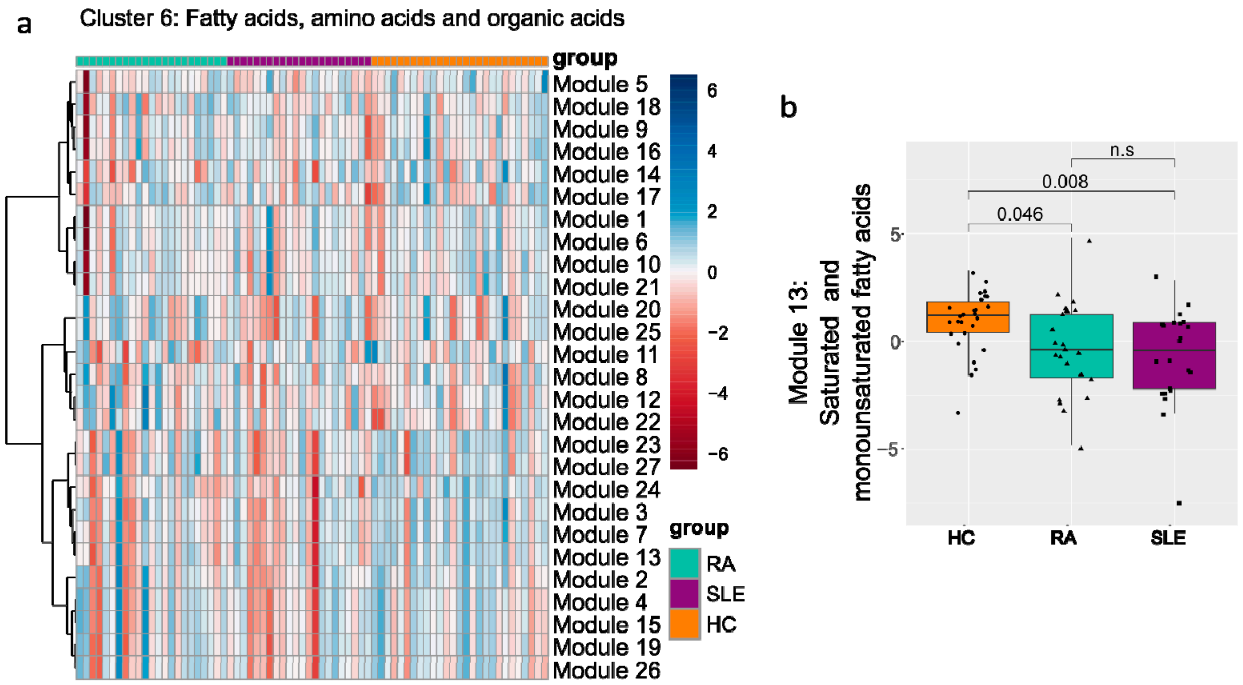
a
Cluster 6: Fatty acids, amino acids and organic acids
group
Module 5
Module 18
Module 9
Module 16
Module 14
Module 17
Module 1
Module 6
Module 10
Module 21
Module 20
Module 25
Module 11
Module 8
Module 12
Module 22
Module 23
Module 27
Module 24
Module 3
Module 7
Module 13
Module 2
Module 4
Module 15
Module 19
Module 26
6
4
2
0
−2
−4
−6
group
RA
SLE
HC
b
Module 13:
Saturated and
monounsaturated fatty acids
n.s
0.008
0.046
5
0
−5
HC
RA
SLE

Figure S4f

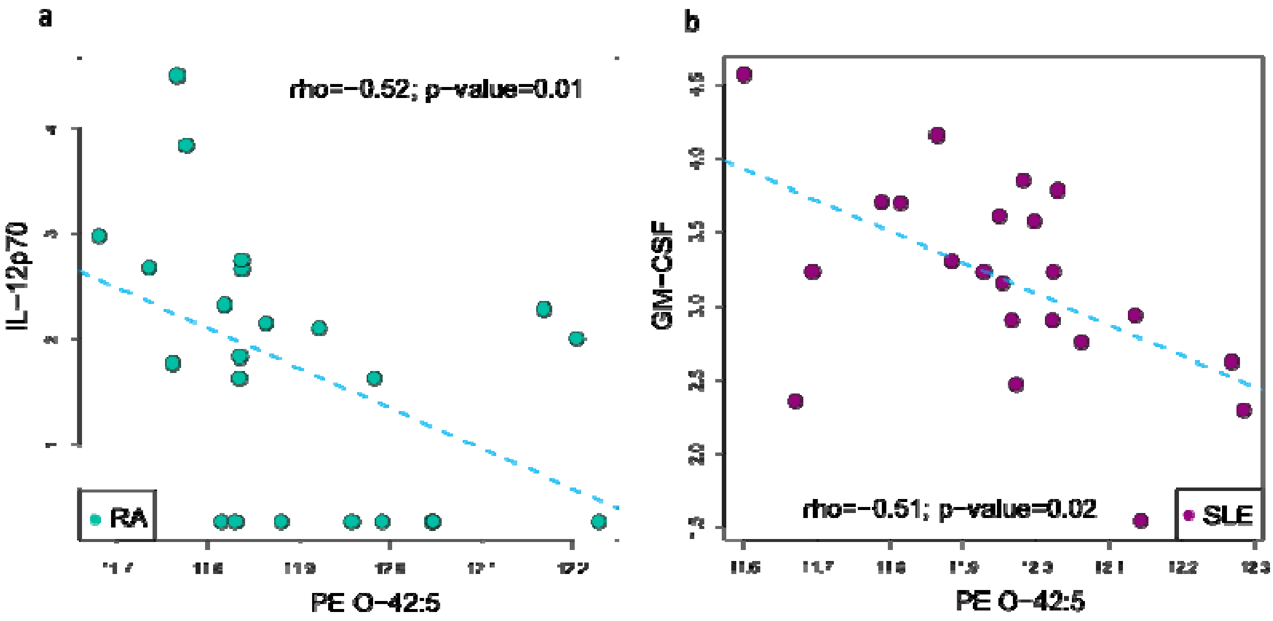

Figure S5. PE O-42:5 is correlated with cytokines in RA and SLE. **a)** Correlation between PE O-42:5 and IL-12p70 in RA patients. **b)** Correlation between PE O-42:5 and GM-CSF in SLE patients

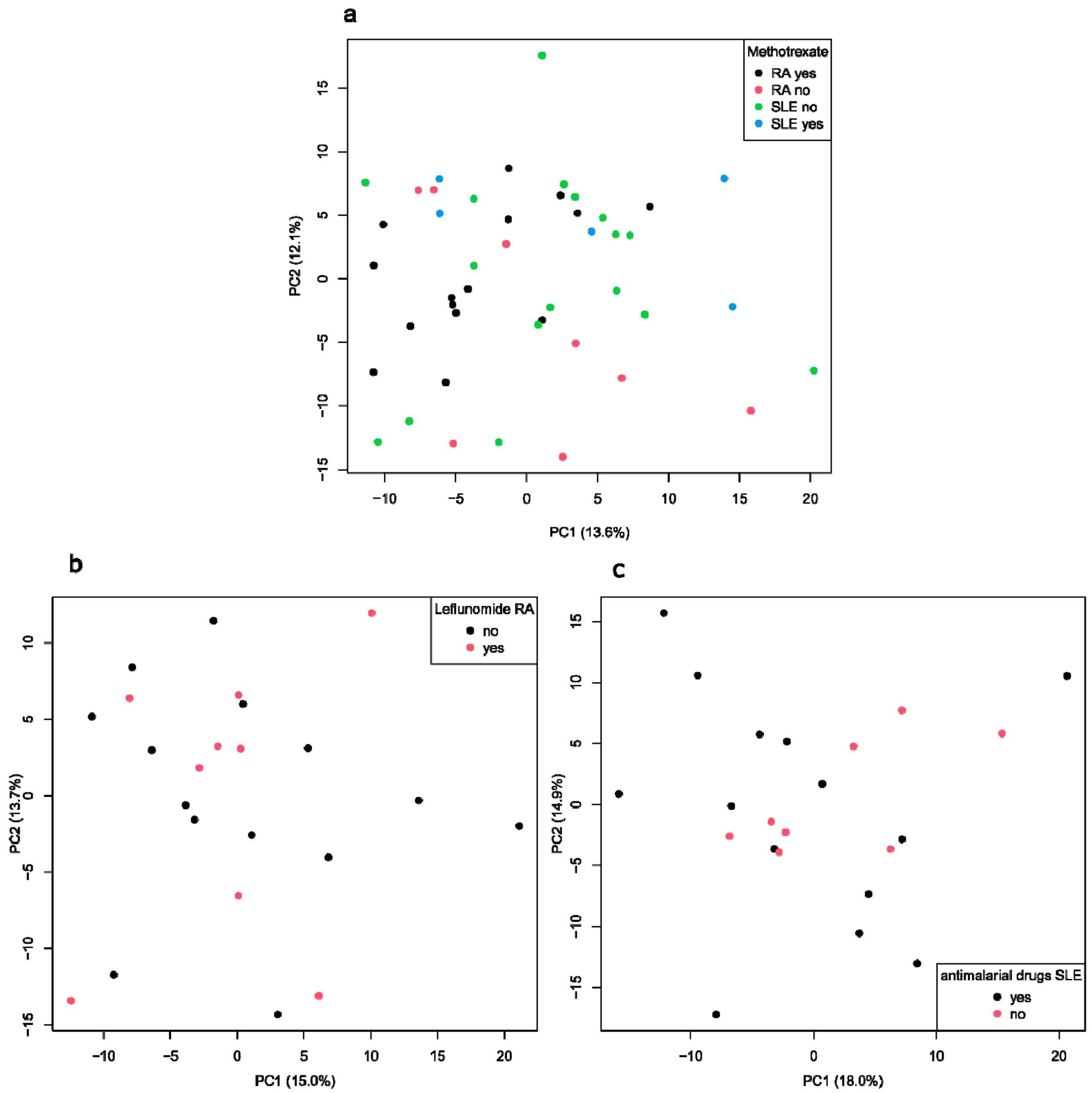

Figure S6. PCA showing the two-dimensional distribution of the RA and SLE patients according to the levels of the metabolites measured. The different plots show the effect of the drug treatments in the two-dimensional distribution of the patients. a) Methotrexate b) Leflunomide c) Antimalarial drugs.
